# Snapshot spectral imaging: from spatial-spectral mapping to metasurface-based imaging

**DOI:** 10.1515/nanoph-2023-0867

**Published:** 2024-03-22

**Authors:** Kaiyang Ding, Ming Wang, Mengyuan Chen, Xiaohao Wang, Kai Ni, Qian Zhou, Benfeng Bai

**Affiliations:** Division of Advanced Manufacturing, Tsinghua Shenzhen International Graduate School, 12442 Tsinghua University, Shenzhen, China; State Key Laboratory of Precision Measurement Technology and Instruments, Department of Precision Instrument, 12442 Tsinghua University, Beijing, China

**Keywords:** snapshot spectral imaging, spatial-spectral mapping, coded modulation, metasurface devices

## Abstract

Snapshot spectral imaging technology enables the capture of complete spectral information of objects in an extremely short period of time, offering wide-ranging applications in fields requiring dynamic observations such as environmental monitoring, medical diagnostics, and industrial inspection. In the past decades, snapshot spectral imaging has made remarkable breakthroughs with the emergence of new computational theories and optical components. From the early days of using various spatial-spectral data mapping methods, they have evolved to later attempts to encode various dimensions of light, such as amplitude, phase, and wavelength, and then computationally reconstruct them. This review focuses on a systematic presentation of the system architecture and mathematical modeling of these snapshot spectral imaging techniques. In addition, the introduction of metasurfaces expands the modulation of spatial-spectral data and brings advantages such as system size reduction, which has become a research hotspot in recent years and is regarded as the key to the next-generation snapshot spectral imaging techniques. This paper provides a systematic overview of the applications of metasurfaces in snapshot spectral imaging and provides an outlook on future directions and research priorities.

## 1 Introduction

The allure of snapshot spectral imaging (SSI) lies in the ability to capture the complete spectral information of a scene in a single “snapshot”, thus enabling real-time dynamic observations, which is particularly suitable for cellular tissue imaging [1], [2], [3], [4], gas diffusion imaging [5], [6], and flame combustion imaging [7], [8]. The ideal SSI system would offer high spatial and spectral resolution while maintaining a compact form factor and computational efficiency. However, achieving this ideal involves tackling a multitude of trade-offs among resolution, capture speed, computational load, and hardware complexity.

At the heart of SSI is the imperative to furnish a comprehensive, accurate representation of the scene’s spectral information. Traditional spectral imaging techniques, such as tunable filters and scanning methods, although effective, are not well-suited for real-time or dynamic scenarios. These methods suffer from the “time-multiplexing dilemma” – the more time taken for capturing spectral data, the less suitable they are for rapidly changing scenes or moving objects.

The quest for better spectral and spatial reconstruction drives recent advancements in SSI. Notable techniques include computational reconstruction methods that use complex algorithms to interpret sensor data, and non-computational methods that rely solely on innovative optical engineering. Each approach has its unique merits and drawbacks in terms of computational efficiency, reconstruction accuracy, and hardware requirements. A particularly promising frontier in SSI is the integration of metasurface technologies, which offer unprecedented control over optical properties at sub-wavelength scales. Metasurfaces can potentially make SSI systems more compact, efficient, and versatile, providing a richer set of degrees of freedom to manipulate incoming light and thereby improve spectral information capture.

In this review, we survey the current state of development of SSI technology based on the differences in data acquisition schemes and detector capture data modes for imaging systems. We first categorize the existing techniques into non-computational spatial-spectral mapping approaches and computationally required coded reconstruction methods, as well as the latest metasurface imaging approaches, summarizing the optical principles, mathematical models, and optical modulation devices necessary for each technique. Finally, we highlight outstanding issues and provide an outlook on future developments, especially in the context of emerging metasurface technologies.

## 2 Spatial-spectral mapping spectral imaging

**Figure 1:**
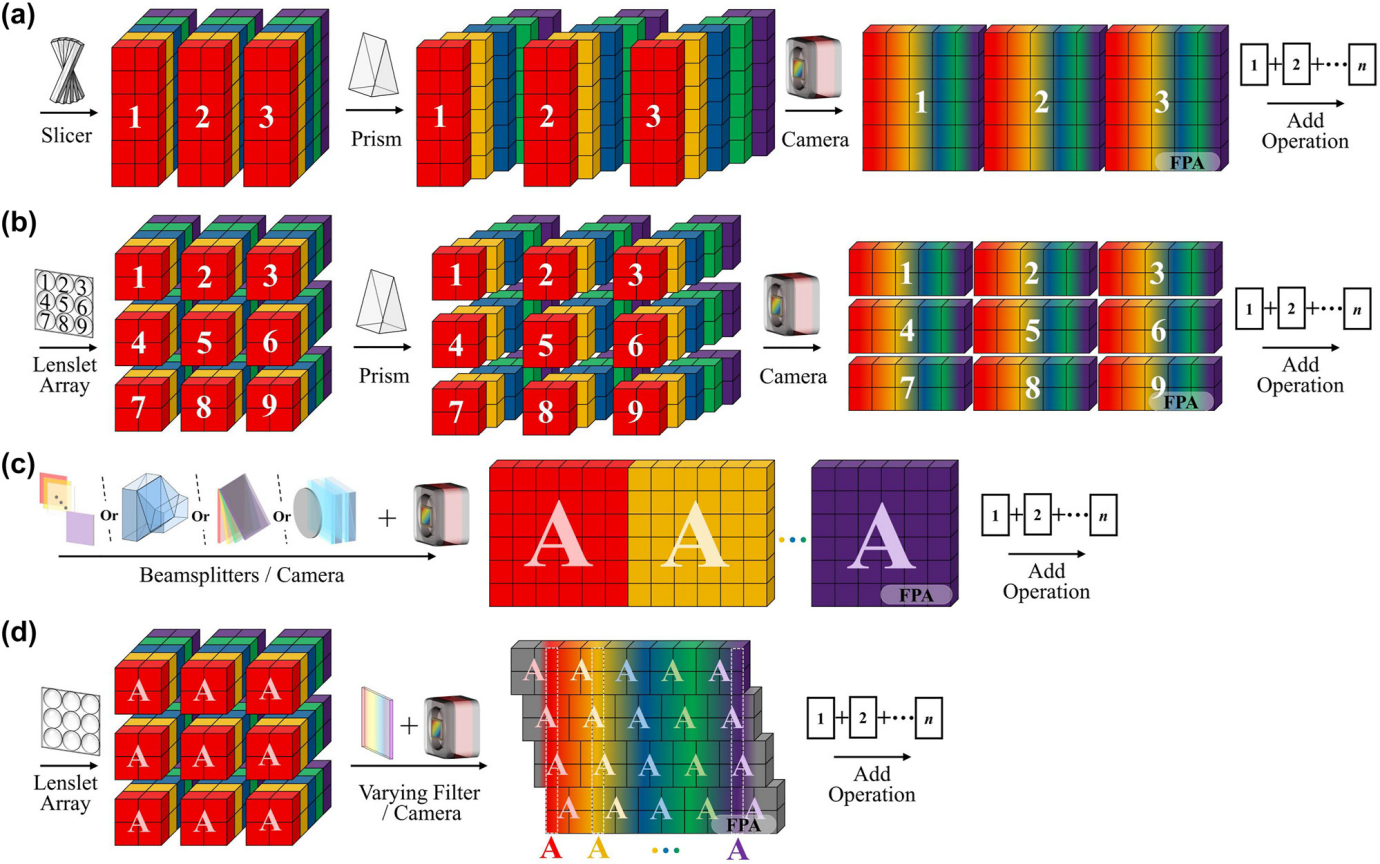
Data modulation scheme for spatial-spectral mapping spectral imaging, where the numbers “1–9” on the cube represent the fields being integrated and the letter “A” illustrates the spatial scene being replicated. (a and b) SSI with slicer mirrors (a) and lenslet array (b) as integral field units. (c and d) Channel-divided (c) and aperture-divided (d) spatial replication SSI techniques.

The spatial-spectral mapping, that is, the spatial-dimensional image or sub-image of the target 3D spectral cube is tiled on the 2D detector along the spectral dimension, resulting in a one-to-one correspondence between the detector pixels and the cube voxels, and the reconstruction can be completed without the subsequent complex algorithm. The fast imaging allows it to be applied to highly dynamic multi-spectral scenarios, such as astronomical nebula observation, biological tissue analysis, and gas diffusion monitoring [9], [10]. However, since the number of detector array pixels limits the observed spectral cube voxels, such instruments involve spatial and spectral resolution tradeoffs, and need to be optimized according to the scene. The mapping type spectral snapshot imager mainly includes integral field [Figure 1(a and b)] and spatial replication types [Figure 1(c and d)].

### 2.1 Integral field snapshot spectral imaging

The integral field spectrometer (IFS) is a 3D spectral imager developed gradually in the late 1980s, initially mainly for astronomical spectral observations, such as TIGER [11], the first scientific IFS telescope system. Compared with the traditional whiskbroom or pushbroom spectrometer, the IFS adopts the integral field unit (IFU) array instead of the slit, which significantly improves the optical throughput and can capture the weak light spectral features, and is widely used *in vivo* fluorescence imaging, astronomical remote sensing, and other applications. The IFU continuously cuts the target image into several sub-images, and then reorganizes the sub-images for focus imaging after dispersion. The complete spectral data cube can be obtained by taking a single photo, and fast imaging ensures the ability of dynamic spectral imaging. The IFU greatly affects the spatial and spectral resolution of the IFS, excessive size reduces detector efficiency, while being too small increases crosstalk between each unit images. Therefore, much research has been invested in the optimization design of IFU to obtain better imaging performance. Currently, the IFU can be divided into three main categories: slicer mirrors, lenslet array and optical fiber bundle.

#### 2.1.1 Slicer mirrors

**Figure 2:**
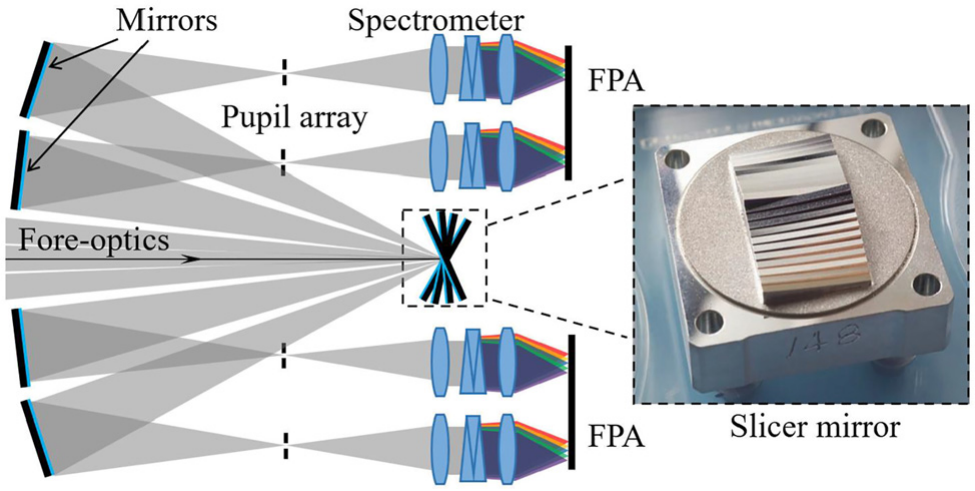
Slicer-based IFS (The Slicer mirror shown was manufactured by Prof. Paul Shore).

The slicer-based spectrometer is the earliest integral field technology and has been applied to various telescope systems [12]. As shown in Figure 2, it usually consists of a front telescopic system, a slicer mirror, a mirror array, and a collimating spectroscopy system. The slicer mirror, which can be made from a series of reflective elements bonded together at different rotation angles or by ultra-precise micromachining technology [12], cuts the rectangular field of view (FOV) of the fore-optics system into a micro-FOV array and reflects the beam into the pseudo-pupil and then into the spectrometer.

The slicer-based spectrometers have a simpler structure than lenslets/fiber optics and do not require complex data post-processing. With good optical design and mechanical assembly, these spectral imagers can efficiently utilize the detector arrays to their fullest potential. In order to obtain better spectral resolution, it is often necessary to increase the size of the instrument to obtain a longer light propagation distance after dispersion. The reported MUSE telescope can achieve a resolution better than 3000 (*R* = *λ*/Δ*λ* is the spectral resolution in a telescopic system), but the size of its entire optical system reaches nearly 3 m in length [13]. In addition, the main drawbacks of such instruments are the high precision required for slicer mirror processing, the optical quality of the contoured surfaces can be as high as 5 nm rms [12], and the narrow slice aperture of the slicer stack can cause severe diffraction (pupil elongation in the direction of dispersion) in the system, resulting in crosstalk [14]. To solve this problem, Zhang et al. adopted spherical slicer mirrors for interference correction to improve spectral resolution [15], [16]. In addition, Content et al. proposed the microslice technique based on the previous research results [17], [18]. The core device is a cylindrical microlens array, that cuts the aperture into rectangular arrays. It transforms the stretched and magnified target images into slit image arrays, and then makes the data cube voxels in the form of narrow bands on the detector. In fact, the principle of the microslice and the pinhole [19] is similar to the lenslet-based spectrometer, which will be described in detail below.

#### 2.1.2 Lenslet array

**Figure 3:**
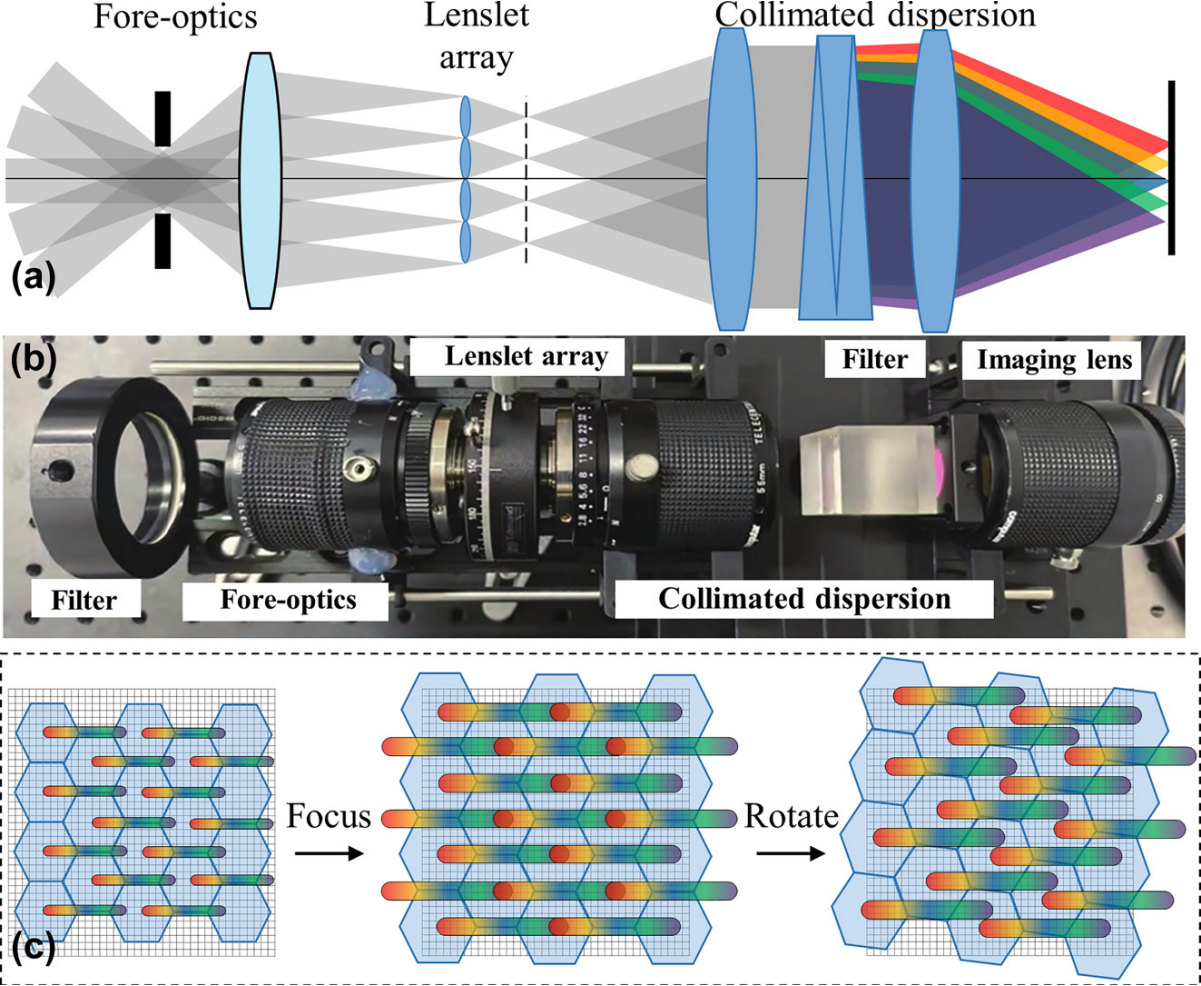
Lenslet-based IFS. (a) Schematic diagram of the optical layout. (b) Experimental setup [25]. (c) Principle of spatial-spectral resolution tunability.

The lenslet-based IFS was first used in astronomy, the OH-Suppressing Infra-Red Imaging Spectrograph in the KECK telescope, the Gemini Planet Imager in the Gemini-south telescope and the Infra-Red Imaging Spectrograph in the Thirty Meter Telescope (under construction) all utilize lenslet arrays [20], [21], [22]. Due to its low-light imaging ability, the lenslet-based technique has also been gradually developed in biological microscopic imaging [23]. As shown in Figure 3(a), the fore-optics imaged the acquired target information $f\left(x,y,\lambda \right)$ onto the lenslet array. The process can be described as

(1) $$\begin{array}{c} f_{1}\left(x,y,\lambda \right)=\underset{m,n}{\sum }rect\left(\frac{x-x_{m}}{D},\frac{y-y_{n}}{D}\right)f\left(x,y,\lambda \right), \end{array}$$

where $f_{1}\left(x,y,\lambda \right)$ is the spectral cube data after lenslet segmentation, $\left(x_{m},y_{n}\right)$ represents the center coordinate of the $\left(m,n\right)$ sub-image, and *D* is the diameter of the lenslet.

Then, the dispersion process can be described as

(2) $$\begin{array}{c} f_{2}\left(x,y,\lambda \right)=\underset{m,n}{\sum }rect\left(\frac{x-x_{m}}{D},\frac{y-y_{n}}{D}\right)f\left(x,y,\lambda \right)p\left(\lambda \right), \end{array}$$

where $p\left(\lambda \right)$ is the transfer function of the dispersive element. In general, lenslet arrays are often rotated at a certain angle to avoid spectral aliasing after sub-image dispersion, and the image distribution on the detector is

(3) $$I\left(x,y\right)=\underset{m,n}{\sum }rect\left(\frac{x-x_{m}}{D},\frac{y-y_{n}}{D}\right)f\left(x,y,\lambda \right)p\left(\lambda \right)r\left(\alpha \right),$$

where $r\left(\alpha \right)$ is the rotation transformation matrix, and optimized designs can be made to align adjacent spectral bands tangentially, making full use of the detector pixels. The lenslet-based spectrometers can be designed with tunable resolution without replacing any optics, and the work of Ji et al. demonstrates a system capable of tunable 0.82–4.17 nm spectral resolution [24]. Figure 3(c) illustrates the tuning process.

The varifocal lens can adjust the size of the sub-image on the detector, according to the formula

(4) $$d=a\frac{f_{f}}{f_{c}},$$

where *d* and *a* are the diameters of the sub-image and pupil image, respectively, *f*_*f*_ and *f*_*c*_ are the collimation and focusing lens focal lengths, respectively.

The spectral band dispersion on the detector can be expressed as

(5) $$\frac{dx}{d\lambda }=f_{f}\frac{d\theta }{d\lambda },$$

where *dθ*/*dλ* represents the angular dispersion ability of the dispersive element. An increase in *dx*/*dλ* leads to image dispersion, resulting in spectral overlap.

To eliminate spectral overlap, the lenslet array needs to be rotated, with a final spectral resolution of

(6) $$\frac{d\lambda }{dp}=\frac{d}{\frac{dx}{d\lambda }}=\frac{a}{f_{c}}\frac{d\lambda }{d\theta }\propto \frac{1}{f_{c}}.$$

Although the raw data acquired by the detector appears to be aliased, after spectral calibration, $p\left(\lambda \right)$ and $\left(x_{m},y_{n}\right)$ in Eq. (3) can be determined, which in turn allows for the establishment of a correspondence between the detector image elements and the cube voxels, and the reconstruction can be completed by looking up the table.

#### 2.1.3 Optical fiber bundle

Lenslet arrays are often used in combination with fiber bundles to directly couple the pupil image into the fiber core. This approach addresses problems related to optical information and energy loss in the fiber-base IFS, arising from incomplete filling caused by the fiber cladding. Consequently, a high optical throughput is achieved [26], making these spectral imagers are widely applicable in astronomical observation.

**Figure 4:**
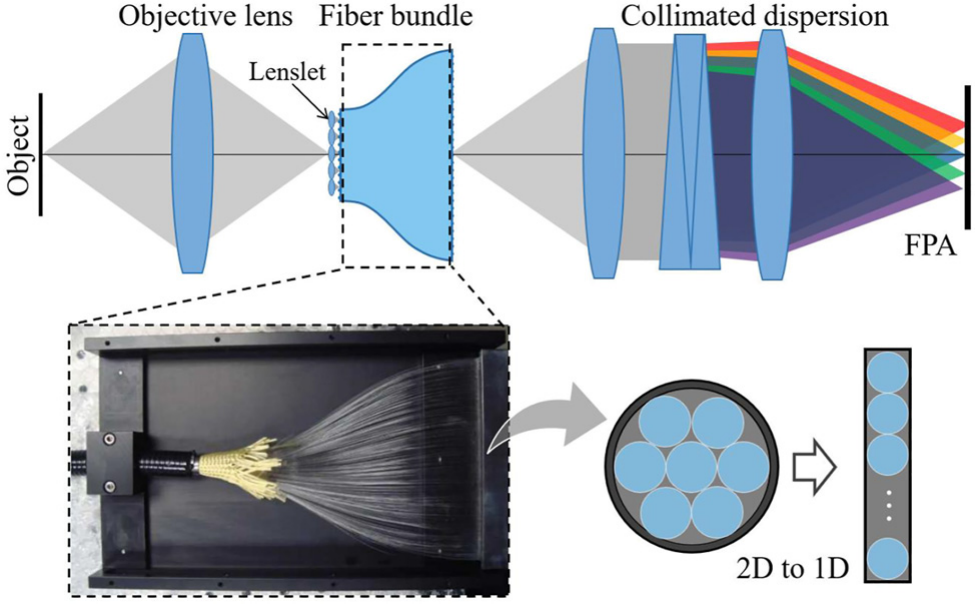
Fiber-based IFS, the subgraph (bottom) is the fiber bundle output [27].

As shown in Figure 4, unlike the previous two IFSs, the optical fiber bundle converts the sub-image array from 2D to 1D, presenting a different image format on the detector [27]. Therefore, there is a high degree of design freedom to adjust the spatial sampling of the system to fit the spectral sampling space and improve the detector pixel utilization to meet specific application requirements. Advances in fiber preparation technology have driven the development of highly integrated fibers, effectively improving spatial resolution. This improvement not only leads to more compact instruments, but also offers potential for the development of medical endoscopy [28], [29], [30].

In general, integral field imaging spectrometers utilize conventional grating or prism devices for spectroscopy, and the optical range of the system after dispersion determines its operating bandwidth and spectral resolution to a certain extent. In order to improve the imaging resolution of the system, it is necessary to increase the optical range length on the one hand, and increase the number of detectors or effective pixels on the other hand, which inevitably leads to an increase in the complexity or overall size of the system, and thus numerous researches have been focused on the innovative optical design to improve the imaging quality. Currently, the IFS has also been combined with cutting-edge technologies and has shown exciting results. Examples include combining compressive sensing for spectral imaging, ultrafast burst imaging, and optical fabrication for novel IFUs [31], [32], [33].

### 2.2 Spatial replication snapshot spectral imaging

Spatial replication spectrometers typically use either a lenslet array (LA) or beam splitters (BS) to perform parallel spatial operations on the target scene. Combined with multispectral filter elements for spectral scanning, these spectrometers generate a set of spectrally separated images on the detector. This setup allows for snapshot imaging through multiple simultaneous sampling.

Depending on the type of optical components employed, spatial replication spectrometers can be divided into two main types: multi-channel beam-splitting spectral imaging and multi-aperture divided spectral imaging.

#### 2.2.1 Multi-channel beam-splitting

**Figure 5:**
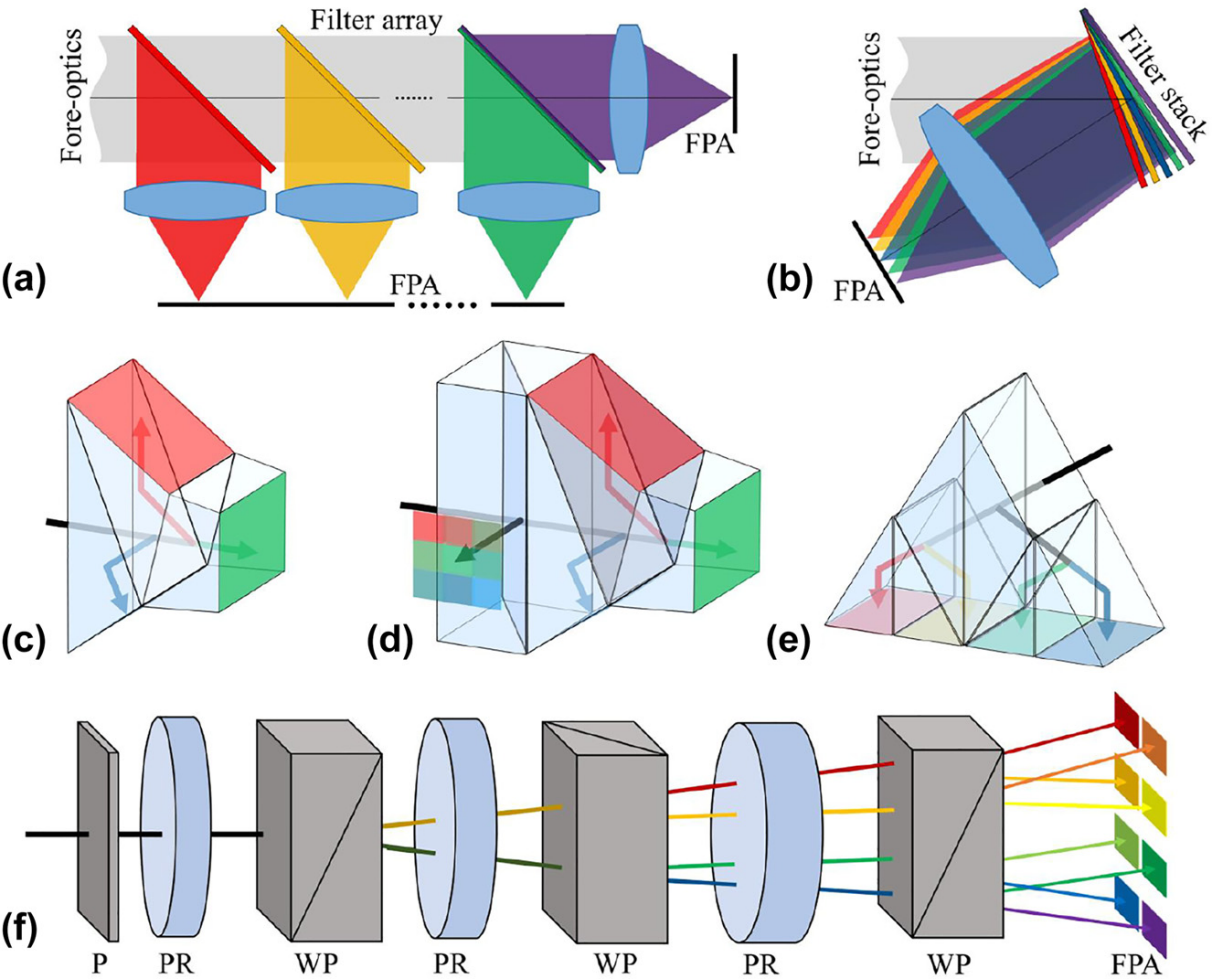
Various types of multispectral beam splitter layouts. (a) Spectral filter array. (b) Spectral filter tilt stack. (c–e) Multichannel prism blocks. (f) Wollaston polarizing beam splitter, P, polarizer; PR, waveplate; WP, Wollaston polarizers.

The primary component in multichannel beam-splitting spectral imaging is the dichroic filter, specifically designed to either reflect or transmit specific light beams. Dichroic filter arrays have a historical presence, dating back to the 1950s when they were employed in television cameras [34]. In this setup, each beam was received by a different detector, as shown in Figure 5(a). To ensure that the multispectral data received by a single detector, the dichroic filter stack composed of slanted filters was also designed [35], see Figure 5(b).

Subsequently, Lang et al. proposed a prism assembly [36] [Figure 5(c)], consisting of three prism blocks. This assembly utilizes total internal reflection and dichroic filters to achieve the separation of spectral channels. Based on their work, Murakami et al. proposed a four-channel separation prism combined with a filter array to achieve mixed-resolution multi-spectral imaging [37] [Figure 5(d)]. In this configuration, one path is received through a multispectral filter array, and higher-resolution spectral reconstruction is performed on this detector data.

In recent years, Greiner et al. and Rothhardt et al. designed a new four-channel BS based on the Kösters prism for space observation [38], [39]. As shown in Figure 5(e), this design uses a dichroic filter and the total reflection at the outer edge surfaces to reflect all four generated beams onto the detector. Additionally, the three prisms in the system allow different assembly layouts to adjust the arrangement of the different band images on the detector. Compared to other designs, the novel Kösters-type BS is more compact and does not require multiple separate detectors.

Harvey et al. [40] introduced enhancements to the traditional Lyot filter by incorporating a Wollaston beam splitting polarizer [Figure 5(f)] in place of the original polarizer to separate the incident beam. This modification, combined with the filtering function of a Lyot birefringent crystal, achieves multi-spectral imaging. Theoretically, if the system adopts *N* cascaded birefringent interference units, 2*N* spectral images can be simultaneously imaged on the detector. However, the system’s operating bands are severely limited by the wavelength-dependent dispersion of Wollaston prisms. As a remedy, it becomes necessary to employ different crystal media or introduce a polarization grating for achromatic performance [41], [42], [43].

Obviously, the most distinctive feature of such imaging systems is the cascading of multiple beam-splitting devices to achieve spectral separation in multiple channels. However, the loss of energy and the increase in volume significantly limit the number of spectral channels that can be obtained.

#### 2.2.2 Multi-aperture divided

Multi-aperture divided spectral imaging usually uses a lenslet array to realize spatial replication, combined with multi-spectral filters to realize spectral cube measurement within a single snapshot.

Hirai et al. [46] used this method to design a multi-image Fourier transform spectrometer. This spectrometer utilized a Michelson interferometer with a tilted mirror to generate position-dependent optical path differences for the interference sub-images on the detector. Subsequent 3D spectral reconstruction is achieved by establishing a Fourier transform relationship between the interferogram and the spectrum.

**Figure 6:**
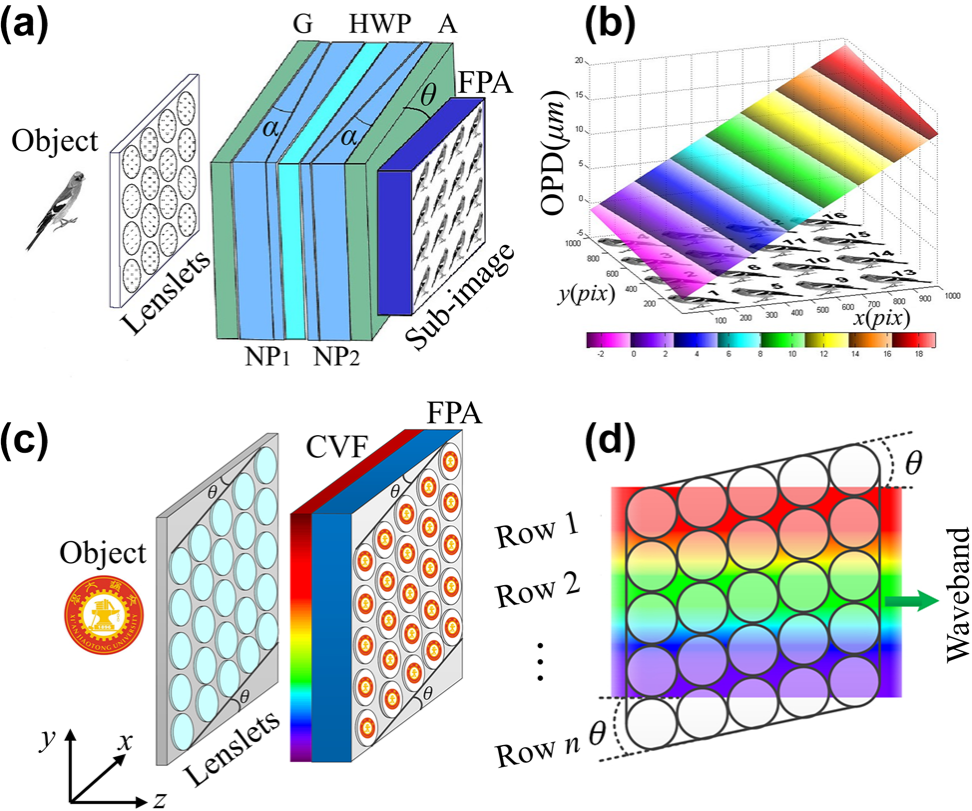
Multi-aperture divided spectral imaging system. (a) Fourier transform snapshot imaging spectrometer [44], two Nomarski prisms (NP) and a half-wave plate (HWP) between them form a birefringent polarization interferometer (BPI). (b) Schematic of the spatial position of the linear optical path differences (OPD) with relation to each sub-image. (c) Schematic of the ORRIS system [45]. (d) Lenslet arrays are arranged tilted to enable spectral scanning of individual sub-images with linear variable filter (LVF). (a and b) Reproduced with permission [44]. Copyright 2012, Optica Publishing Group. (c and d) Reproduced with permission [45]. Copyright 2019, Optica Publishing Group.

Similarly, Kudenov et al. [44] replaced the Michelson interferometer with a birefringent Nomarski prism to construct a more compact and vibration-resistant snapshot spectrometer, as shown in Figure 6(a and b). The rotation angle of the prism relative to the detector is deliberately designed, allowing the sub-images to have distinct optical path differences. Spectral reconstruction is then accomplished by the Fourier transform.

Hubold et al. [47] pioneered the imaging scheme involving a tilted LVF combined with LA, as shown in Figure 6(c and d). This approach not only reduces fabrication difficulty and cost but also introduces challenges in balancing the constraints between detector wastage and spectral perturbation due to the LVF tilt angle. In 2019, Mu et al. [45] introduced a modification by tilting the LA instead of the LVF, significantly reducing the spectral perturbations and ensuring stability for efficient detector utilization.

The mechanisms employed by both approaches to achieve simultaneous multisampling of the continuous spectrum are rather similar. Replicated individual spatial sub-images are scanned by different continuous spectra, resulting in complete spatial-spectral data due to the angle between the filter and the detector. However, the size of the LA determines the maximum number of spectral channels that can be realized by the system, and the spatial dimensional replication implies an average distribution of the number of spatial dimensional pixels, where an increase in spectral resolution inevitably leads to a decrease in spatial resolution.

## 3 Coded reconfiguration spectral imaging

**Figure 7:**
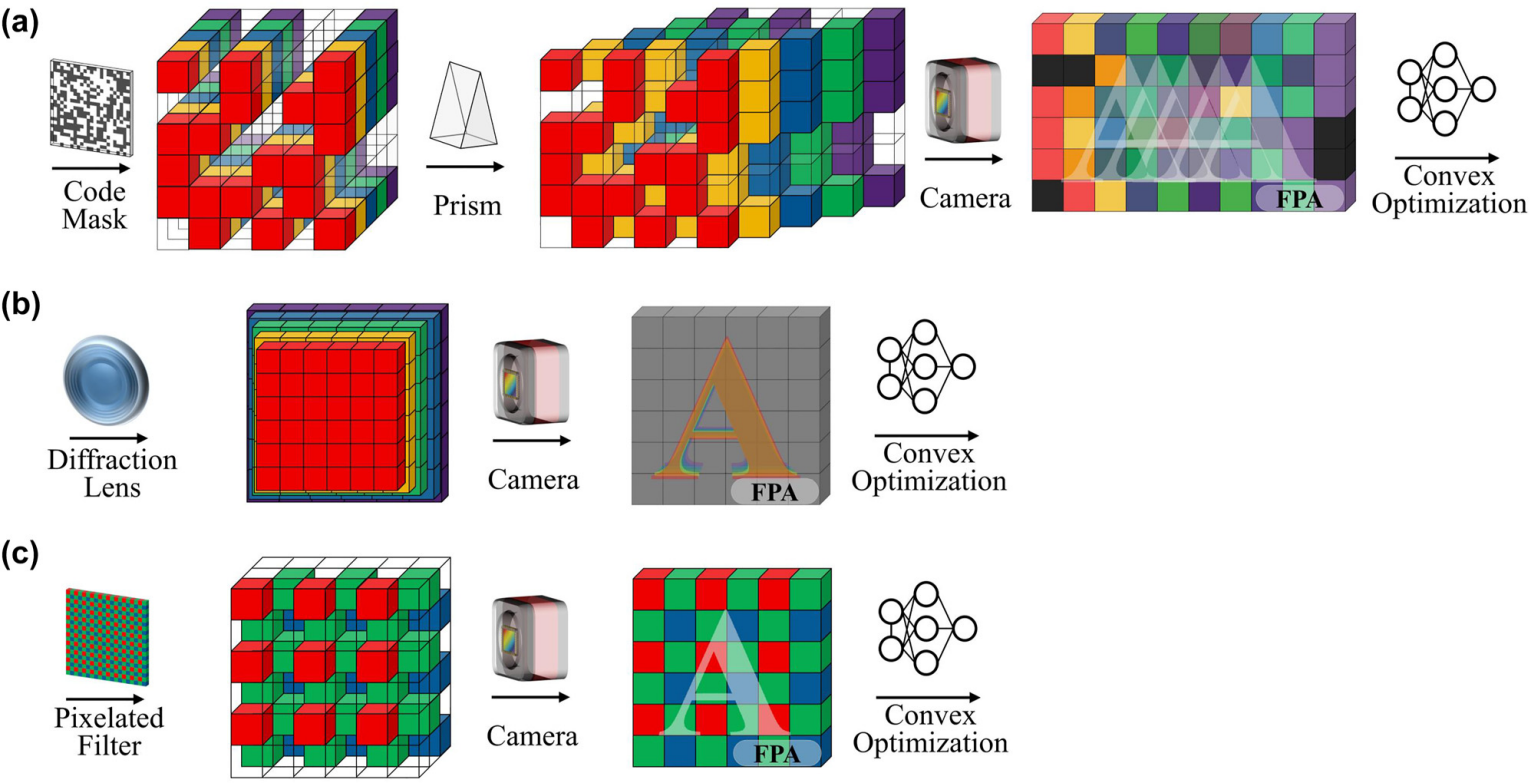
Data modulation scheme for coded reconfiguration spectral imaging. (a) Coded aperture snapshot spectral imaging (amplitude encoding). (b) Snapshot spectral imaging based on diffractive optical elements (phase encoding). (c) Snapshot spectral imaging based on pixelated filter arrays (wavelength encoding).

Coded reconfiguration spectral imaging involves encoding the amplitude, phase, or wavelength information of light using specially designed optics. The encoding process can be expressed as a sensing matrix for the system, allowing the establishment of a mathematical model. This, combined with the theory of compressed sensing, enables the reconstruction of a spectral cube, as depicted in Figure 7. Computational spectral imaging is expected to overcome the limitations of traditional methods, achieving higher resolution data with an equal number of detector pixels and enabling more efficient measurements.

However, such spectral imaging systems rely on precise calibration and demand substantial computational resources. Currently, there are relatively few practical applications, with the only commercially available choice being pixelated wavelength-encoded snapshot spectral cameras [48].

### 3.1 Coded aperture spectral imaging

**Figure 8:**
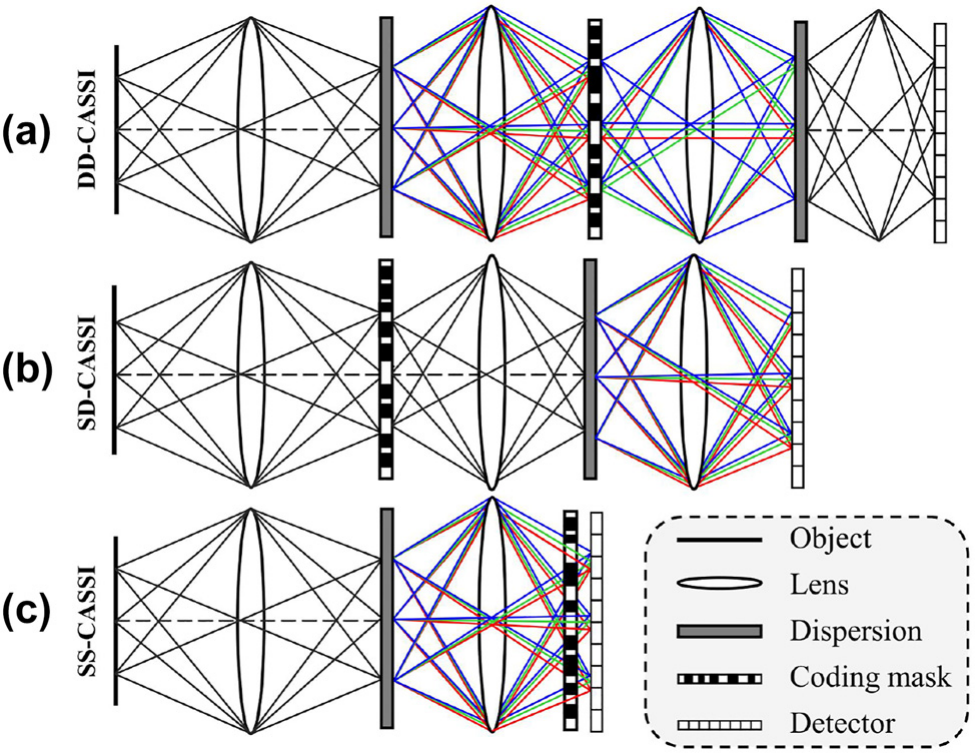
Optical layout diagram of the CASSI system. (a) DD-CASSI with the coding mask located between the two dispersion devices. (b) SD-CASSI, the coding mask is located before the dispersion device and simultaneously modulates the spatial-spectral data. (c) SS-CASSI with the coding mask located some distance in front of the detector.

The common spatial modulation devices are randomly distributed binary coding masks. According to their different positions in the optical path, we can divide the coded aperture snapshot spectral imaging (CASSI) into three categories: spectral coding with double-dispersion, spatial coding with single-dispersion, and spatial-spectral coding, corresponding to DD-CASSI, SD-CASSI, and SS-CASSI, respectively [Figure 8(a–c)]. In addition, there are grayscale coding masks instead of 0–1 coding, and color coding masks combined with multispectral filters.

#### 3.1.1 DD-CASSI

Brady et al. first proposed a snapshot compressive imaging spectrometer using a coded aperture with a double-dispersion structure (DD-CASSI) in 2006 [49], [50]. This structure uses two dispersive elements for spectral splitting and combining, respectively, with the coded aperture placed in the middle of the system to enable sparse sampling of spectral information. As shown in Figure 8(a), a target $f\left(x,y,\lambda \right)$ is dispersed spectrally after passing through a dispersive element, which can be expressed as

(7) $$f_{1}\left(x,y,\lambda \right)=f\left(x-\alpha \left(\lambda -\lambda_{0}\right),y,\lambda \right),$$

where *α* is the grating dispersion rate, *λ*_0_ is the initial wavelength. The lens focuses the dispersed target information onto the coding mask of size Δ for modulation:

(8) $$\begin{array}{ll} f_{2}\left(x,y,\lambda \right) & =f_{1}\left(x,y,\lambda \right)t\left(x,y\right)\\ t\left(x,y\right) & =\underset{m,n}{\sum }t\left(m,n\right)\text{rect}\left(\frac{x}{\Delta }-m,\frac{y}{\Delta }-n\right), \end{array}$$

where *t* is 0 or 1, indicating the transmission pattern of the coding aperture, (*m*, *n*) represents the corresponding coding sub-aperture. A subsequent lens system recombines the dispersed spectral information again:

(9) $$f_{3}\left(x,y,\lambda \right)=f_{2}\left(x+\left(\lambda -\lambda_{0}\right),y,\lambda \right),$$

and the final lens system focuses the encoded target scene onto the focal plane array (FPA) with a spectral response of $r\left(\lambda \right)$ :

(10) $$\begin{array}{c} I\left(x,y\right)=\int f\left(x,y,\lambda \right)t\left(x+\alpha \left(\lambda -\lambda_{0}\right),y\right)r\left(\lambda \right)\text{d}\lambda . \end{array}$$

Due to the sparse sampling process and the superposition of light from different object points and wavelengths, the amount of measurement data is much smaller than the original data cube, and thus often needs to be reconstructed from the measurement data by reconstruction algorithms. To do this, we discretize Eq. (10) as the following:

(11) $$I_{i,j}=\underset{k}{\sum }F_{i,j,k}T_{i,j+k-1,k}R\left(\lambda_{k}\right),$$

where $F\in R^{M\times N\times L}$ is the scene data cube, *M* × *N* is the spatial dimension, *L* is the spectral dimension, and the coded aperture transmittance is $T\in {\left[0,1\right]}^{\left(M+L-1\right)\times N\times L}$ .

The design of double-dispersion can effectively utilize the detector, but it also leads to the use of too many optical elements, which makes it require a high assembly precision. In 2021, Reflective DD-CASSI [51], [52] proposed by Yu et al. used a reflective coded aperture and a folded optical path to obtain a compact structure and realized two dispersion processes using only one dispersive element, but the system optical throughput was reduced due to the use of beam splitter mirrors.

#### 3.1.2 SD-CASSI

In single-dispersion CASSI (SD-CASSI) [53], only one dispersive element is used, the scene light is first spatially sampled by the coded aperture before passing through the dispersive element, and the detector accepts the integral of the individual spectral images after misalignment. Therefore, SD-CASSI requires a larger detector with a smaller coded aperture than DD-CASSI. Similarly, the data obtained at the detector can be expressed as

(12) $$I_{i,j}^{\prime }=\sum F_{i,j-k+1,k}^{\prime }T_{i,j-k+1,k}^{\prime }R\left(\lambda_{k}\right)+N_{i,j}^{\prime }.$$

In addition, Cao et al. proposed a system with a more simplified structure [54], which consists only of a coded mask, a prism, and a grayscale camera. The well-designed mask samples the space so that the spectra of each sampling point do not overlap each other on the detector, and can be used to acquire spectral video in real time without reconstruction algorithms, at the expense of spatial resolution. Instead of static binary encoded apertures, some scholars have used a digital micromirror device (DMD), whose rotation angle of each micromirror can be individually controlled at high speeds, to enable encoded apertures for grayscale values [55], [56], [57], which provides an improvement in reconstruction accuracy.

#### 3.1.3 SS-CASSI

In 2014, Lin et al. proposed a dual-coding system design [58], where the scene light is first focused onto a DMD for spatial coding, then dispersed by a diffraction grating, and finally spectrally modulated by a liquid crystal on a silicon display. This scheme increases the cost and requires additional calibration. They later improved the scheme [59] by placing the coding aperture of the SD-CASSI some distance before the detector [Figure 8(c)] to achieve spatial-spectral co-modulation, and the experimental results were significantly better than those of the SD-CASSI. In addition, Rueda et al. also proposed a dual-coding system [60], which introduces two high-resolution coding templates with different transmittances to encode the spatial-spectral information, but it requires multiple measurements to realize the high-resolution spectral reconstruction.

### 3.2 Pixelated filter array spectral imaging

Pixelated filtered spectral imaging enables wavelength coding by transmitting or reflecting a specific spectrum through a periodic arrangement of pixel-level units directly deposited or monolithically integrated on a detector. This allows the simultaneous acquisition of spatial and spectral information. The imaging process can be expressed as

(13) $$\begin{array}{ll} I\left(x,y\right) & =f\left(x,y,\lambda \right)T\left(x,y,\lambda \right)\\ & =f\left(x,y,\lambda \right)\underset{i,j}{\sum }t_{i,j}\left(\lambda \right)rect\left(\frac{x}{B}-i,\frac{y}{B}-j\right), \end{array}$$

where $T\left(x,y,\lambda \right)$ denotes the filter array transmittance function, $\left(i,j\right)$ is the coordinate of the sub-filter.

In addition, there is a diversity of structural designs of pixel-level filter units, aiming at more wavelength bands, higher transmittance, and more cost-effective spectral imaging. According to the different fabrication methods they can be classified as: color pigment filters, Fabry–Pérot filters and sub-wavelength patterned grating filters (as well as metasurface filters, which will be discussed in Section 4).

#### 3.2.1 Color filter array

**Figure 9:**
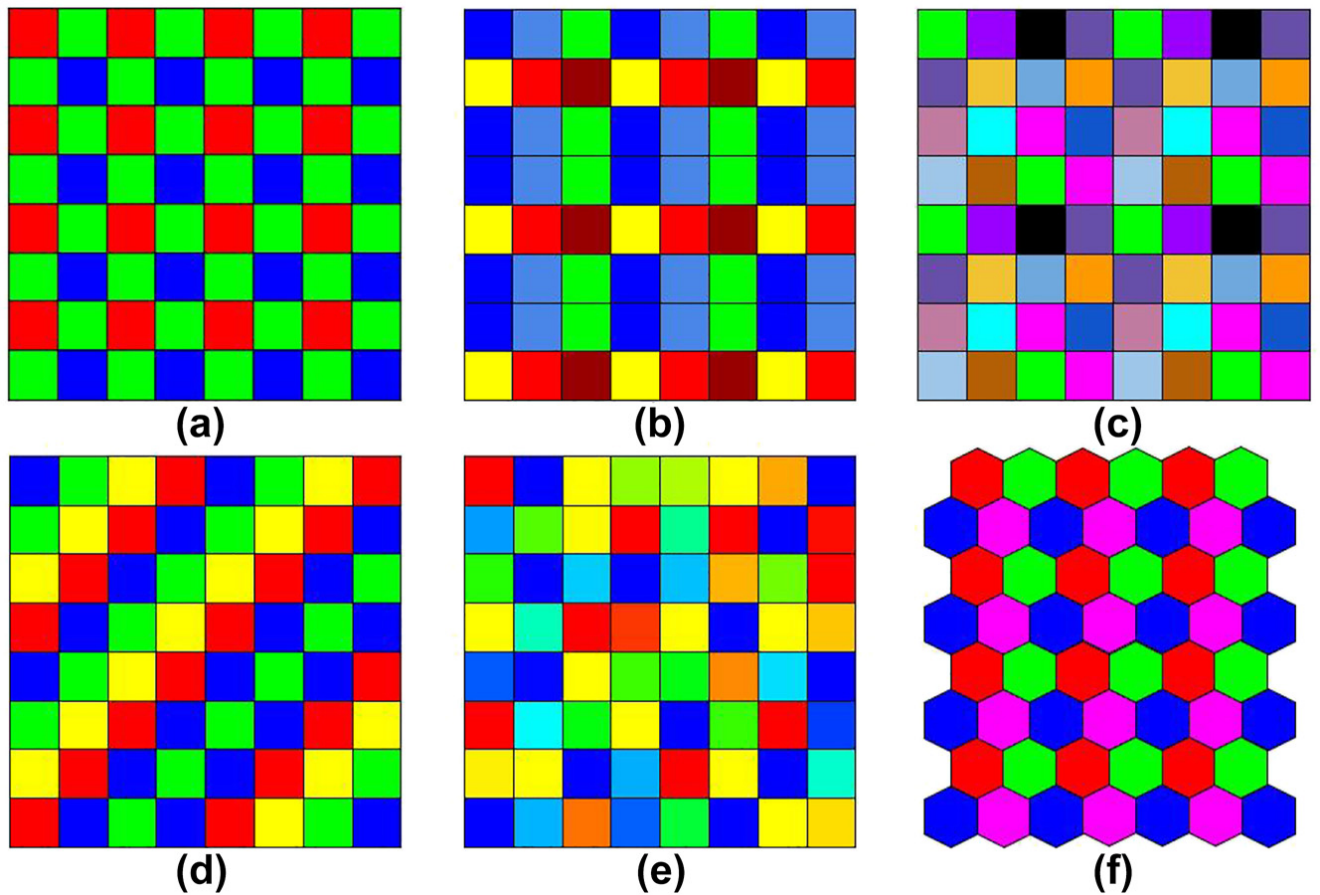
Different multispectral filter array (MSFA) designs [64]. (a) Bayer 2 × 2 array. (b–c) 3 × 3 and 4 × 4 MSFA. (d–e) Uniformly and randomly distributed MSFA. (f) MSFA with hexagonal pixels.

Bayer arrays [Figure 9(a)] have been widely used in modern photographic equipment since they were proposed in 1976 [61]. It consists of 2 × 2 three primary color filters, where different colors are achieved by transmitting (absorbing) layers of organic or pigment dyes [62]. Employing interpolation algorithms, it is possible to obtain an RGB image from a Bayer image that matches the vision of the human eye. Nowadays, the application of hyperspectral data makes the reconstruction of spectral information from RGB images of interest to researchers [63].

Additionally, increasing the number of filter bands is the most direct and effective way to improve the imaging quality. Similar to RGB cameras, multispectral cameras based on the division-of-focal-plane (DoFP) technique usually carry four or more channels of filters, which are designed for spatial uniformity and spectral consistency [65] to form a multispectral filter array [Figure 9(b and c)], so that multispectral data can be acquired in a single shot [64], [66]. However, this sacrifices spatial resolution to some extent, and missing information at different locations and bands needs to be filled in by demosaicing algorithms [67], [68].

To facilitate digital storage and processing, almost all MSFAs use square pixels, but hexagonal pixels [Figure 9(f)] have also been reported [69]. Most multispectral cameras based on DoFP technology combine filters of different wavelength bands into hyperpixels, which are then aligned on the FPA [64]. Aggarwal et al. proposed two different alignment patterns: uniform [Figure 9(d)] and random [Figure 9(e)] distribution [70], where randomly distributed MSFAs can be reconstructed as spectral images by compression-aware algorithms.

#### 3.2.2 Fabry–Pérot filter array

**Figure 10:**
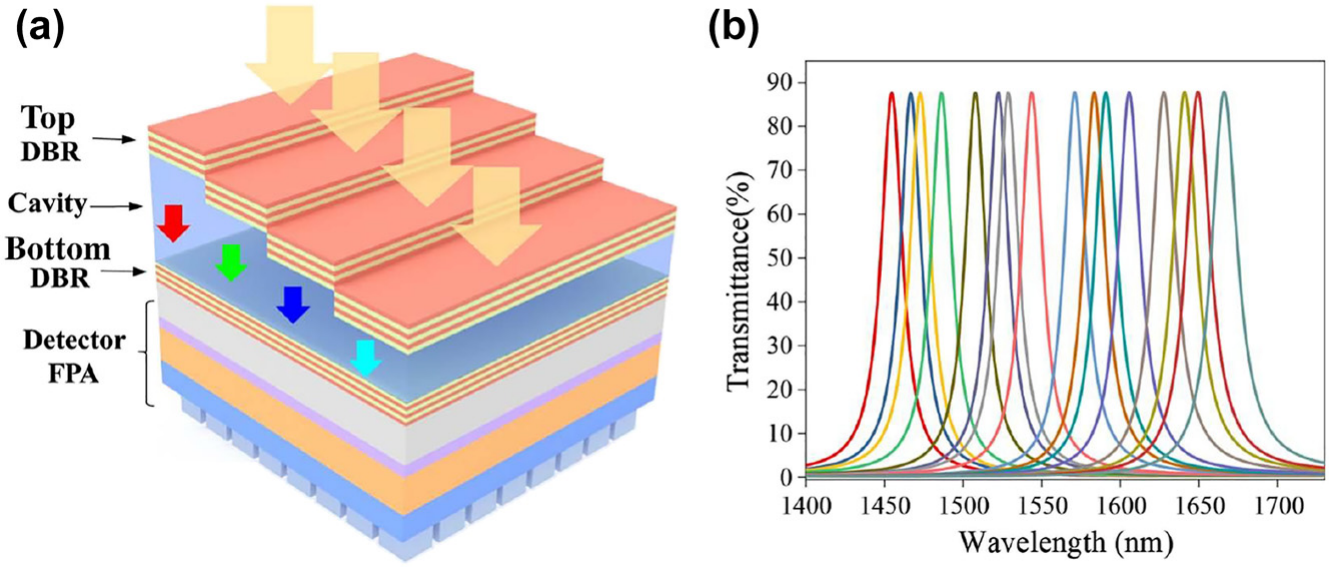
Schematic diagram of the integrated F–P filter [71]. (a) 3D structure of a multilayer F–P filter. (b) Transmission spectrum of the filter array. Reproduced with permission [71]. (a and b) Copyright 2022, Chinese Laser Press.

Fabry–Pérot (F–P) MSFA can be obtained by integrating multiple micro F–P filters that allow the transmission of a single wavelength, and it is usually necessary to fabricate multiple F–P microcavities of different lengths to produce a series of different narrow-band optical transmissions, as shown in Figure 10.

In 2004, Correia et al. [72] designed an arrayed FP spectrometer with 16 bands by varying the thickness of SiO_2_ deposited on CMOS, overcoming previously developed F–P spectrometers with limited bands and the need for displacement actuation. Almost concurrently, Wang et al. successively proposed dielectric-type structure and dual-cavity structure FP filters [73], and realized spectral imaging with more bands by improving the lithography [74] and deposition processes [75]. Currently, most of the related researches focus on the efficient or high-precision fabrication of cavity layers with different thicknesses (nanoimprinting [76], electron beam lithography [77], laser direct write lithography [78]).

#### 3.2.3 Subwavelength patterned grating filter array

Conventional diffraction gratings are often used as dispersive devices for spectral imaging. The phenomena of guided mode resonance or surface plasmon resonance allow well-designed sub-wavelength patterned gratings to be used as narrow-band filters [79], [80], [81], [82]. Thus, subwavelength patterned gratings customized by varying grating parameters (thickness, period, duty cycle) can provide significant transmission for specific wavelengths, and pixelated grating filter arrays can be integrated with sensors for snapshot spectral imaging.

**Figure 11:**
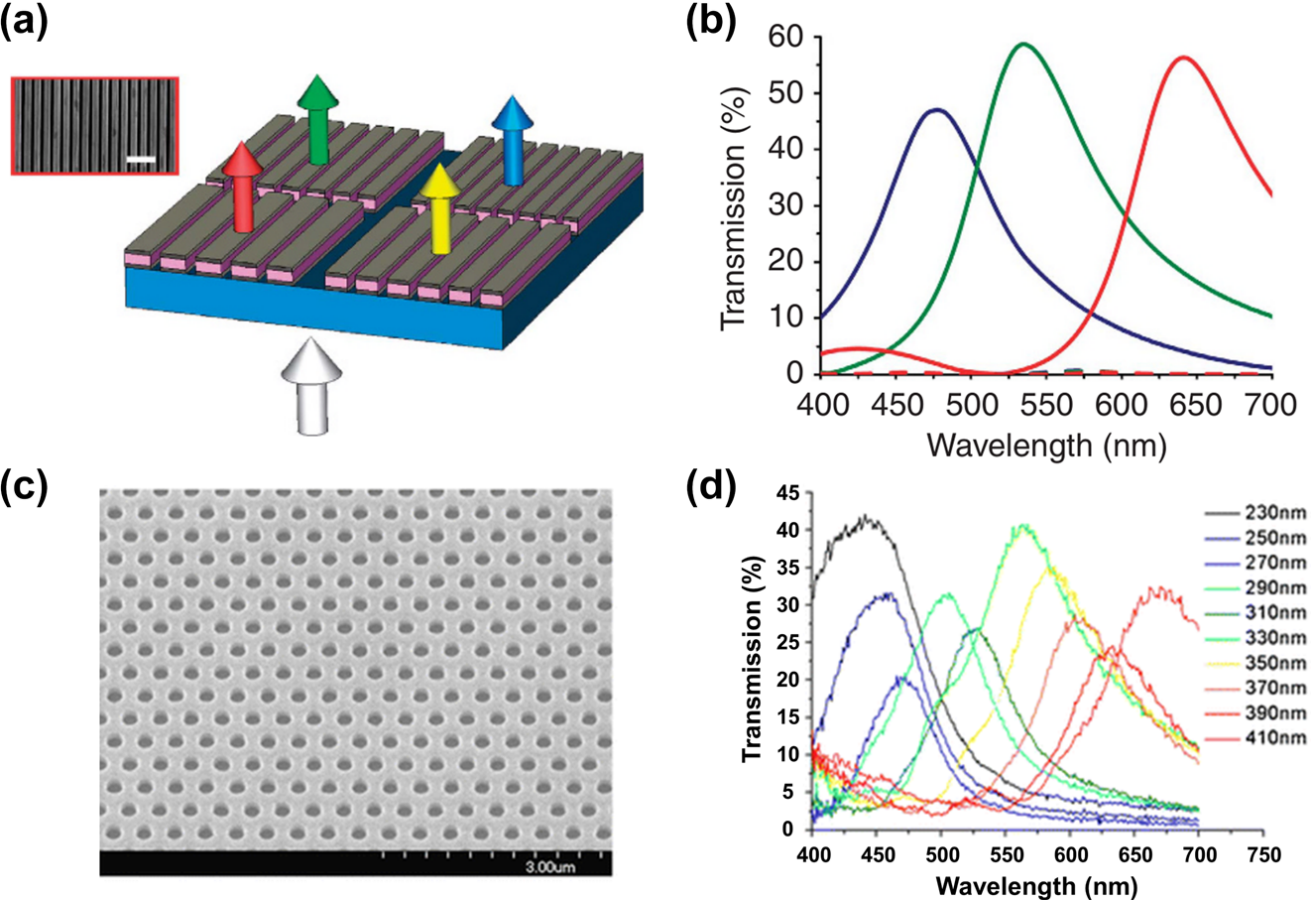
Patterned grating color filters [82], [84]. (a) Schematic diagram of the plasmonic nanoresonators. (b) Simulated transmission spectra for the RGB filters. (c–d) SEM image and transmissive spectra of two-dimensional hole array plasma filters. (a and b) Reproduced with permission [82]. Copyright 2010, Nature Publishing Group. (c–d) Reproduced with permission [84]. Copyright 2012, Springer.

In 2010, Haïdar et al. [83] fabricated a mid-wave infrared filter array with 10 wavelength bands using symmetric, freestanding sub-wavelength metallic gratings, and this grating structure with a narrow slit theoretically enables near-perfect optical transmission, as shown in Figure 11(a and b).

However, considering the polarization-sensitive nature of one-dimensional gratings, some scholars have begun to design two-dimensional sub-wavelength structures. Chen et al. [84] proposed periodic sub-wavelength hole arrays, which achieve transmission at different wavelengths by changing the period of the cells, see Figure 11(c and d). This plasma color filter was directly integrated into the sensor using electron beam lithography and dry etching engraving to achieve panchromatic sensitivity in the visible band of 100 × 100 pixels. Subsequently, numerous similar arrays of sub-wavelength hole structures have been proposed for high-performance filtering in different wavelength bands [85].

Delving further, these studies, in conjunction with the field of surface plasmonics, have given rise to a cutting-edge scientific discipline known as metasurfaces. This emerging frontier offers novel avenues for advancements in spectral imaging.

### 3.3 Diffraction modulation spectral imaging

A frequently employed modulator in spectral imaging is the diffractive optical element (DOE), a phase modulator that introduces an additional phase change to the light field by altering the shape of the DOE. This alteration allows for the acquisition of a wavelength-dependent point spread function (PSF), enabling the distinction of light at various wavelengths. With different types of DOEs, phase-encoded spectral imaging can be categorized into following three groups.

#### 3.3.1 Diffractive diffuser

**Figure 12:**
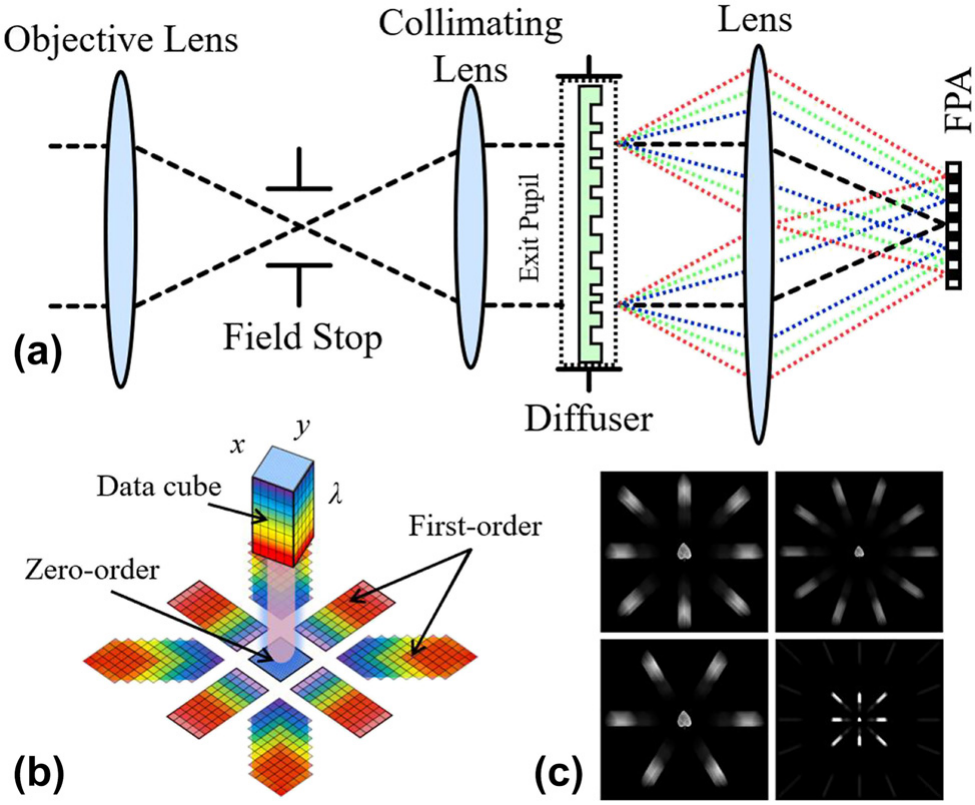
The computed tomography imaging spectrometer. (a) System optical layout. (b) Schematic diagram of the computed tomography imaging spectrometer [90]. (c) Multiple diffraction pattern image simulation [91]. (b) Reproduced with permission [90]. Copyright 2023, Optica Publishing Group.

The earliest diffraction spectral snapshot imaging was the computed tomography imaging spectrometer (CTIS) proposed by Okamoto et al. in 1991 [86], which has been gradually developed and applied to biomedical, astronomical, agricultural and other fields [87], [88], [89]. In this system [Figure 12(a)], the object $f\left(x,y,\lambda \right)$ passes through the diffraction grating (can be regarded as a diffuser) and is collected by the FPA:

(14) $$I\left(x,y\right)=\overset{\infty }{\underset{0}{\int }}r\left(\lambda \right)\left[f\left(x,y,\lambda \right)*p\left(x,y,\lambda \right)\right]d\lambda ,$$

where * denotes convolution, $r\left(\lambda \right)$ is the spectral response of the detector, and $p\left(x,y,\lambda \right)$ is the PSF of the CTIS system, which can be expressed as

(15) $$p\left(x,y,\lambda \right)=\overset{I}{\underset{i=-I}{\sum }}\overset{J}{\underset{j=-J}{\sum }}a_{ij}\delta \left(x-i\frac{\lambda f}{d},y-j\frac{\lambda f}{d}\right),$$

where $\delta \left(\cdot \right)$ is the Dirac delta function, *I*, *J* is the maximum diffraction order, *a*_*ij*_ is the corresponding diffraction efficiency, *f* is the focal length of the imaging lens, and *d* is the grating constant.

The raw data on the FPA can be represented as

(16) $$I\left(x,y\right)=\overset{I}{\underset{i=-I}{\sum }}\overset{J}{\underset{j=-J}{\sum }}a_{ij}\overset{\infty }{\underset{0}{\int }}r\left(\lambda \right)f\left(x-i\frac{\lambda f}{d},y-j\frac{\lambda f}{d},\lambda \right)d\lambda .$$

As shown in Figure 12(b), the acquired image contains projections of the data cube along multiple angles, where the 0th order of diffraction is equivalent to the integration of the cube data along the spectral axis on the FPA. The remaining diffraction orders of light undergo diffraction for wavelength-dependent spatial shifts, resulting in dispersion in all directions.

Additionally, numerous studies have focused on improving the spectral resolution, and diffraction gratings with different projections [92], [93], [94], [95] have been designed [Figure 12(c)] to improve the performance of CTIS, taking into account the spatial spectral trade-off, FPA factor, and missing cone angle [96], [97].

**Figure 13:**
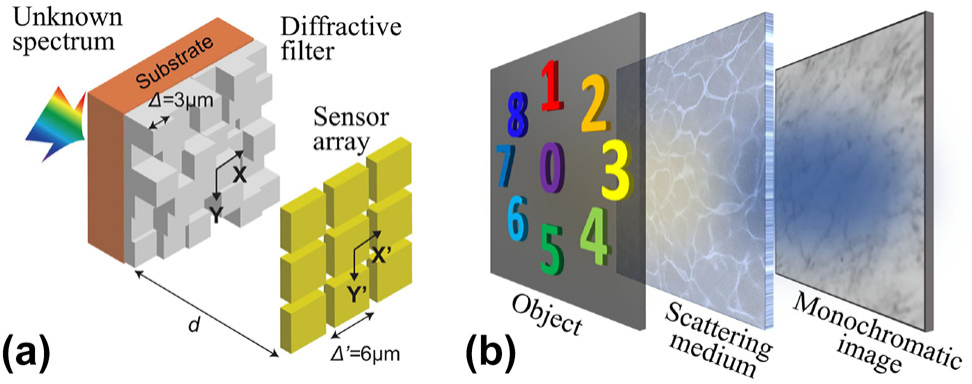
Schematic of multi-spectral imaging of scattering medium. (a) Diffuser consisting of a designed array of randomized highly diffractive filters. (b) Light travels through a strong scattering medium and produces a speckle pattern on a monochrome camera.

All the above diffraction grating modulators realize regular discrete phase modulation, and some random phase modulation devices have also been used for spectral imaging, e.g., Wang et al. designed a random discrete phase device [Figure 13(a)], which can be used as a filter for spectral imaging after arraying [98], and Sahoo et al. proposed a random continuous phase modulation device (scattering medium) [Figure 13(b)], which also utilizes the variability of the PSF in different spectral bands to achieve spatial-spectral information modulation [99].

#### 3.3.2 Diffractive lens

Unlike diffractive diffusers, diffractive lenses generally have a rotationally symmetric structure and belong to axial chromatic DOE. They can achieve chromatic imaging without the need for additional lenses, hence they are also referred to as lensless spectral imaging. Their characteristics include being lightweight and compact, with advantages in scalability and field of view.

**Figure 14:**
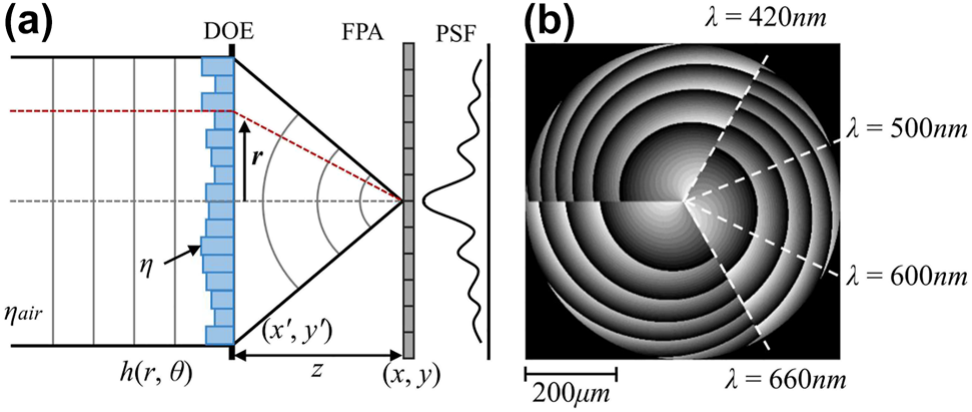
Spectral imaging with diffraction lenses. (a) Imaging via a diffractive lens and its PSF. (b) Diffractive lens designed in [100]. Reproduced with permission [100]. (a and b) Copyright 2019, Association for Computing Machinery.

For a diffractive spectral imaging system [Figure 14(a)], the incident light field with initial phase *ϕ*_0_ and amplitude *A*_0_ at the front surface of the DOE can be expressed as

(17) $$u_{0}\left(x^{\prime },y^{\prime },\lambda \right)=A_{0}{\text{e}}^{\text{i}\phi_{0}\left(x^{\prime },y^{\prime },\lambda \right)}.$$

As the light field passes through the DOE, a phase shift generated by the DOE surface profile $h\left(x^{\prime },y^{\prime }\right)$ will be obtained:

(18) $$\phi_{h}\left(x^{\prime },y^{\prime },\lambda \right)=\frac{2\pi }{\lambda }\left[n\left(\lambda \right)-n_{0}\left(\lambda \right)\right]h\left(x^{\prime },y^{\prime }\right),$$

where $n\left(\lambda \right)$ and $n\left(\lambda_{0}\right)$ denote the refractive index of the DOE and the propagation medium, respectively, and the modulated light field at the rear surface of the DOE is expressed as

(19) $$u_{1}\left(x^{\prime },y^{\prime },\lambda \right)=A_{0}{\text{e}}^{\text{i}\left[\phi_{0}\left(x^{\prime },y^{\prime },\lambda \right)+\phi_{h}\left(x^{\prime },y^{\prime },\lambda \right)\right]}.$$

After Fresnel diffraction [101] it propagates forward a distance *z* to reach the detector, which leads to

(20) $$\begin{array}{ll} u_{2}\left(x,y,\lambda \right) & =\frac{{\text{e}}^{\frac{i2\pi z}{\lambda }}}{i\lambda z}\iint u_{1}\left(x^{\prime },y^{\prime },\lambda \right){\text{e}}^{\frac{i\pi }{\lambda z}\left[{\left(x-x^{\prime }\right)}^{2}+{\left(y-y^{\prime }\right)}^{2}\right]}\text{d}x^{\prime }\text{d}y^{\prime }, \end{array}$$

where $\left(x,y\right)$ represents the coordinates of image space, and the PSF can be obtained from Eq. (19) as

(21) $$p\left(x,y,\lambda \right)\propto {\left|F\left[A_{0}{\text{e}}^{\text{i}\left[\phi_{0}\left(x^{\prime },y^{\prime },\lambda \right)+\phi_{h}\left(x^{\prime },y^{\prime },\lambda \right)+\frac{\pi }{\lambda z}\left(x^{\prime 2}+y^{\prime 2}\right)\right]}\right]\right|}^{2},$$

where $F\left(\cdot \right)$ denotes Fourier transform. If $u_{0}\left(x^{\prime },y^{\prime },\lambda \right)$ is a plane light field, Eq. (21) can be simplified as

(22) $$p\left(x,y,\lambda \right)\propto {\left|F\left[{\text{e}}^{\text{i}\left[\phi_{h}\left(x^{\prime },y^{\prime },\lambda \right)+\frac{\pi }{\lambda z}\left(x^{\prime 2}+y^{\prime 2}\right)\right]}\right]\right|}^{2}.$$

Similarly with Eq. (14), the image obtained on the detector can be expressed as

(23) $$I\left(x,y\right)=\overset{\infty }{\underset{0}{\int }}r\left(\lambda \right)\left[u_{0}\left(x^{\prime },y^{\prime },\lambda \right)*p\left(x,y,\lambda \right)\right]d\lambda .$$

Jeon et al. proposed rotational diffraction snapshot spectral imaging [100], where the height profile of the designed DOE is a three-equal spiral structure, see Figure 14(b), enabling it to form a PSF with a three-wing shape that rotates with the spectral changes. The PSF’s anisotropy and size consistency greatly improve the quality of the spectral reconstruction performed by the devised end-to-end network. Hu et al. extended the work of [100] by realizing a PSF with two wings and achieved similar results [102]. Similarly, Xu et al. [103] improved the DOE design and reconstruction algorithm in [100]. Baek et al. proposed a DOE with a PSF that varies with depth and spectral [104], making it possible to reconstruct spectral and depth from a single captured image. In addition, Li et al. noted that the fabrication of DOEs requires the quantization of the surface height, and better results were obtained by taking the effect of quantization into account in both DOE design and image reconstruction [105].

#### 3.3.3 Diffractive optical network

Diffractive optical networks (or diffractive deep neural networks, D2NN) were first proposed by Lin et al. in 2018, which realize the function of neural networks in an all-optical manner through multiple diffractive surfaces [106]. Li et al. used a trained diffractive network to encode spatial information into feature spectral, realizing the task of terahertz spectral classification under a single-pixel detector, and proving that the diffractive network can be used for multi-wavelength information processing [107].

**Figure 15:**
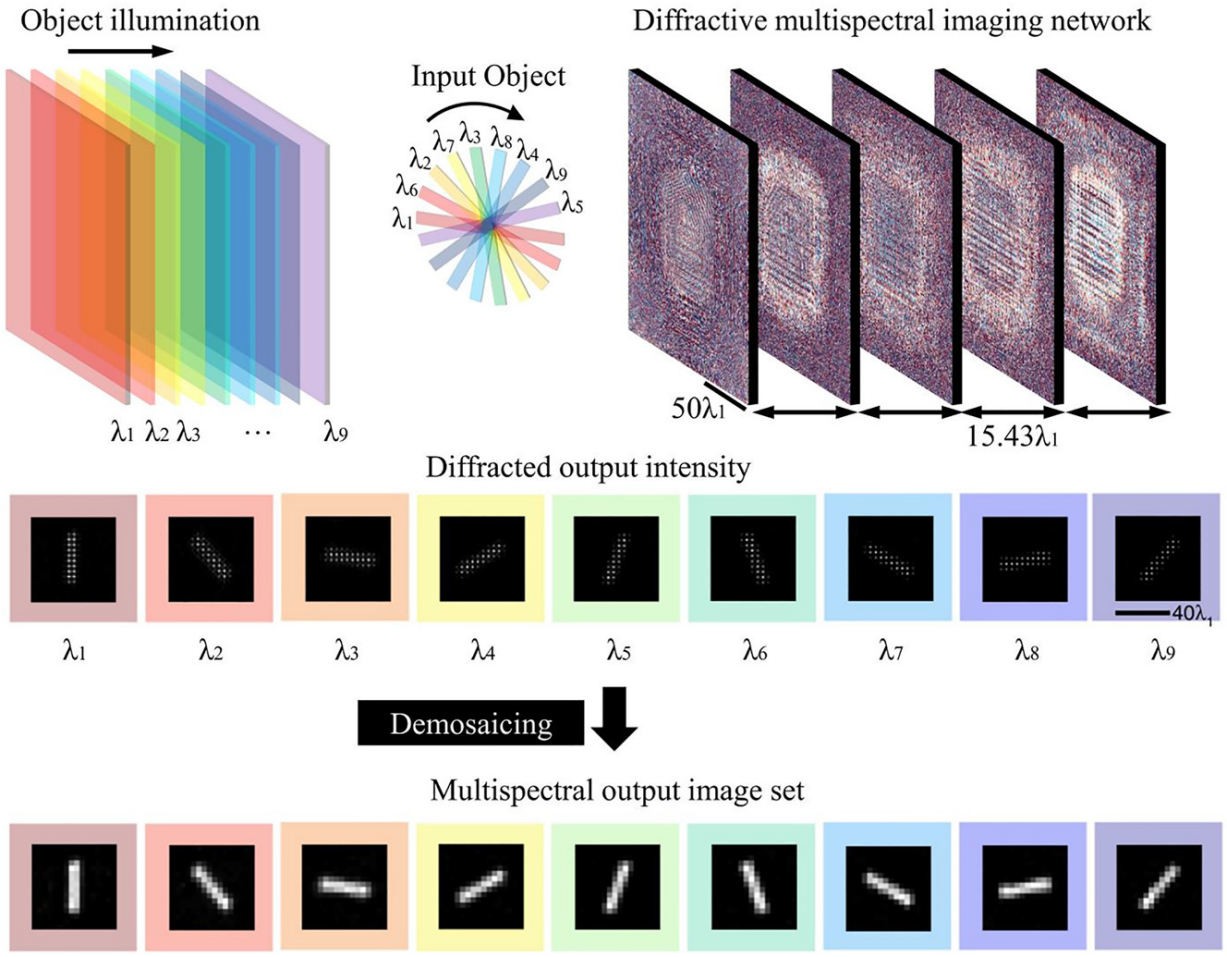
Snapshot spectral imaging based on diffractive optical networks [108]. Reproduced with permission [108]. Copyright 2023, Nature Publishing Group.

In 2023, Mengu et al. [108] introduced a diffractive optical network into multispectral snapshot imaging, as shown in Figure 15. This method performs spatial coherent imaging over a broad spectrum while simultaneously directing a pre-determined set of spectral channels to a pixel array on the output plane. In essence, the approach converts a monochromatic focal plane into a virtual MSFA. Notably, this method eliminates the need for spectral filters or image recovery algorithms.

### 3.4 Combined modulation spectral imaging

In order to enhance the flexibility of modulation and increase the degree of spatial-spectral information multiplexing, some researchers have performed combined modulation to improve the imaging performance, but this also leads to a complex system architecture and additional reconfiguration computational cost.

#### 3.4.1 Amplitude & wavelength modulation

Arce et al. incorporated pixel-level spectral filters into CASSI, significantly enhancing CASSI’s modulatory capabilities in the spectral domain. They argue that the increased modulation depth better satisfies the restricted isometry property (RIP) during the measurement process [109]. They mainly introduced two configurations: The first replaces the binary coded aperture in SD-CASSI with a spectral filter array [109], [110], and the second configuration employs an image sensor based on an MSFA, designed to capture light dispersed by a dispersive element in the scene [111], [112]. The spectral response function at each detector pixel is position-dependent; by rotating the dispersive element in the system, multiple snapshots can be obtained for improved imaging [113]. Additionally, they analyzed the application of RGB image sensors in SD-CASSI and found that they provide superior imaging performance compared to monochromatic sensors [114].

#### 3.4.2 Phase and wavelength modulation

Some researchers combine DOE with sensors based on MSFA to achieve SSI [115], [116]. A spectral diffusercam was proposed in [116], building on earlier work [117], where the PSF was designed to span multiple superpixels. In this configuration, the light received on each superpixel is a composite from various points, thereby achieving compressed imaging. Another work [118] replaced the MSFA-based sensor with a color-coded aperture set at a specific distance from a monochromatic sensor, resulting in a shift-variant PSF. They further discussed the impact of filter distribution on the color-coded aperture on the reconstructed spectral. Kim et al. generated wavelength-dependent PSFs using LVF in combination with phase masks [119] to encode the spectral information with lensless imaging onto a monochrome image sensor.

#### 3.4.3 Amplitude and phase modulation

Kar et al. [120] proposed a diffractive lens compressive spectral imaging, where the coded aperture spatially modulates the light field in the scene and uses a diffractive lens for dispersion and focusing. However, this technique requires multiple measurements to achieve diversity sampling and sparse reconstruction algorithms to recover spectral data.

### 3.5 Spectral reconstruction technologies

Different coded modulation techniques, despite their diverse physical architectures, share a common forward measurement model due to the inherent nature of their compressed measurements. This model can be expressed as the following system of linear equations:

(24) y=Φf+n,

where *y* denotes the system measurement, *f* is the full datacube with size *M* × *N* × *L*, $\Phi \in R^{MN\times MNL}$ is the sensing matrix, and *n* is system noise.

This solution problem is a typical ill-posed problem, which is generally based on two approaches, i.e., solving the convex optimization problem by constructing constraints, or using data-driven deep learning methods to achieve data prediction.

Reconstruction methods based on optimization algorithms directly formulate optimization problems using the system’s forward model and manually chosen prior information. The prior information utilized in constructing optimization problems often draws from methods traditionally employed in image reconstruction and restoration, such as sparse priors [121], smoothness priors [122], low-rank priors [123], etc. Sparse priors assume that the data can be represented in a transformed domain as *f* = Ψ*t*, and the representation in the transformed domain, *t*, exhibits sparsity. This transformation can be the Fourier transform, wavelet transform, and so on. The resulting optimization problem is formulated as

(25) $$\hat{f}=\Psi \left(\underset{t}{\mathrm{argmin}}{\left\|y-\Phi \Psi t\right\|}_{2}^{2}+\tau {\left\|t\right\|}_{1}\right),$$

where the 1-norm is used to constrain sparsity. The smoothness prior is based on the observation that natural images often exhibit a certain degree of smoothness. An optimization problem can be formulated as

(26) $$\hat{f}=\underset{f}{\mathrm{argmin}}{\left\|y-\Phi f\right\|}_{2}^{2}+\tau {\left\|f\right\|}_{\mathrm{TV}},$$

where the regularization term, total variation (TV), calculates the gradient of adjacent pixels in the image, serving as a common metric for smoothness in image reconstruction. The low-rank prior, considering internal data correlations, stems from the fact that natural images tend to have low-rank structures. The optimization problem can be expressed as

(27) $$\hat{f}=\underset{f}{\mathrm{argmin}}{\left\|y-\Phi f\right\|}_{2}^{2}+\tau {\left\|f\right\|}_{*},$$

where the regularization term involves the nuclear norm.

Algorithms for solving these optimization problems include TwIST [124], GPSR [125], GAP-TV [126], and so on. However, these algorithms require iterative computations, resulting in long computation times and limited precision in the solutions. In 2019, Liu et al. proposed a rank-minimization-based method called DeSCI [127] specifically for CASSI. They reported a peak signal-to-noise ratio (PSNR) exceeding 8.27 dB, surpassing previous algorithms. However, the reconstruction process still took several minutes. Due to the manual determination of regularization terms in optimization problems, they may not accurately capture real-world scenarios. This limited generalization makes it challenging to achieve the detailed reconstruction of complex targets.

In recent years, advancements in artificial intelligence technology have led to the development of novel algorithms tailored to diverse physical architectures in SSI. These algorithms, focused on learning general patterns from extensive data, exhibit characteristics such as real-time processing, high reconstruction accuracy, and richer reconstruction details compared to traditional iterative methods [128], [129].

The CASSI system has a simple system structure and thus has received more attention in terms of reconfiguration algorithms. Proposed methods include end-to-end frameworks [130], deep unfolding frameworks [131], and others. The current state-of-the-art (SOTA) model, DAUHST [131], employs a transformer-based deep unfolding approach, reporting a PSNR as high as 38.36 dB. This represents a significant improvement of 15.24 dB over the classical TwIST iterative algorithm.

For narrowband filter array applications, numerous studies are dedicated to recovering spectral images from RGB images [63]. Since this can be considered as three-channel multispectral imaging, spectral reconstruction methods designed for RGB can be extended to multispectral filter arrays. Pixel-wise spectral reconstruction methods, such as manifold learning [132] and basis function fitting [133], attempt to directly recover spectral information from measurements in each channel but overlook the spectral information correlation between pixels. Considering this correlation, patch-wise spectral reconstruction methods can achieve better results. The transformer-based MST++ [134], leveraging the spatially sparse yet spectrally self-similar nature of hyperspectral imaging, has achieved high-precision reconstruction and secured the first position in the NTIRE 2022 Challenge on Spectral Reconstruction from RGB.

For CTIS systems, recent efforts have utilized CNN [91] and GAN [90]-based frameworks for spectral reconstruction, resulting in improvements in speed and accuracy.

## 4 Metasurface-based spectral imaging

**Figure 16:**
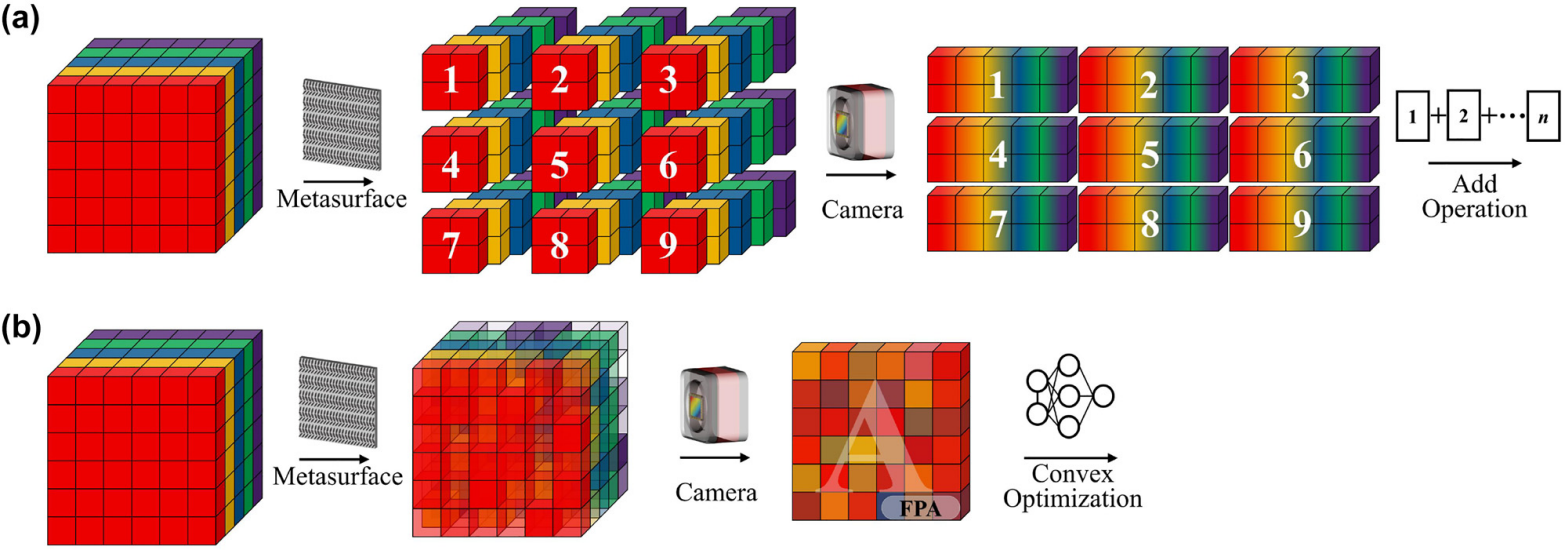
Data modulation scheme for metasurface-based spectral imaging. (a) Using micro-metalens array instead of lenslet array and dispersion devices for field segmentation and dispersion. (b) Using pixelated metasurface substitution to achieve spatial and spectral modulation.

Metasurfaces, comprised of sub-wavelength scale nanostructures, offer a flexible means to modulate optical information, including amplitude, phase, and wavelength of light in an unconventional manner. This flexibility facilitates the design of highly customized and integrated spectral imaging solutions. Figure 16 illustrates both spatial-spectral mapping and coded reconstruction spectral imaging techniques achieved through the incorporation of metasurfaces.

### 4.1 Spatial-spectral mapping with metasurface

Traditional spatial-spectral mapping systems face challenges such as the substantial space occupied by field splitters, spatial replicators, and dispersive elements, along with issues related to optical path alignment. Recent studies have shown that the tremendous design flexibility offered by metasurfaces can substantially replace complex device cascades, achieving multi-functionality in a single device. For example, a comparison between Figures 1(b) and 16(a) illustrates this capability, resulting in ultra-compact, high-performance snapshot spectral imaging. In the following sections, we will introduce the latest applications of metasurface-based SSI aligned with traditional snapshot approaches.

#### 4.1.1 Integral field type

**Figure 17:**
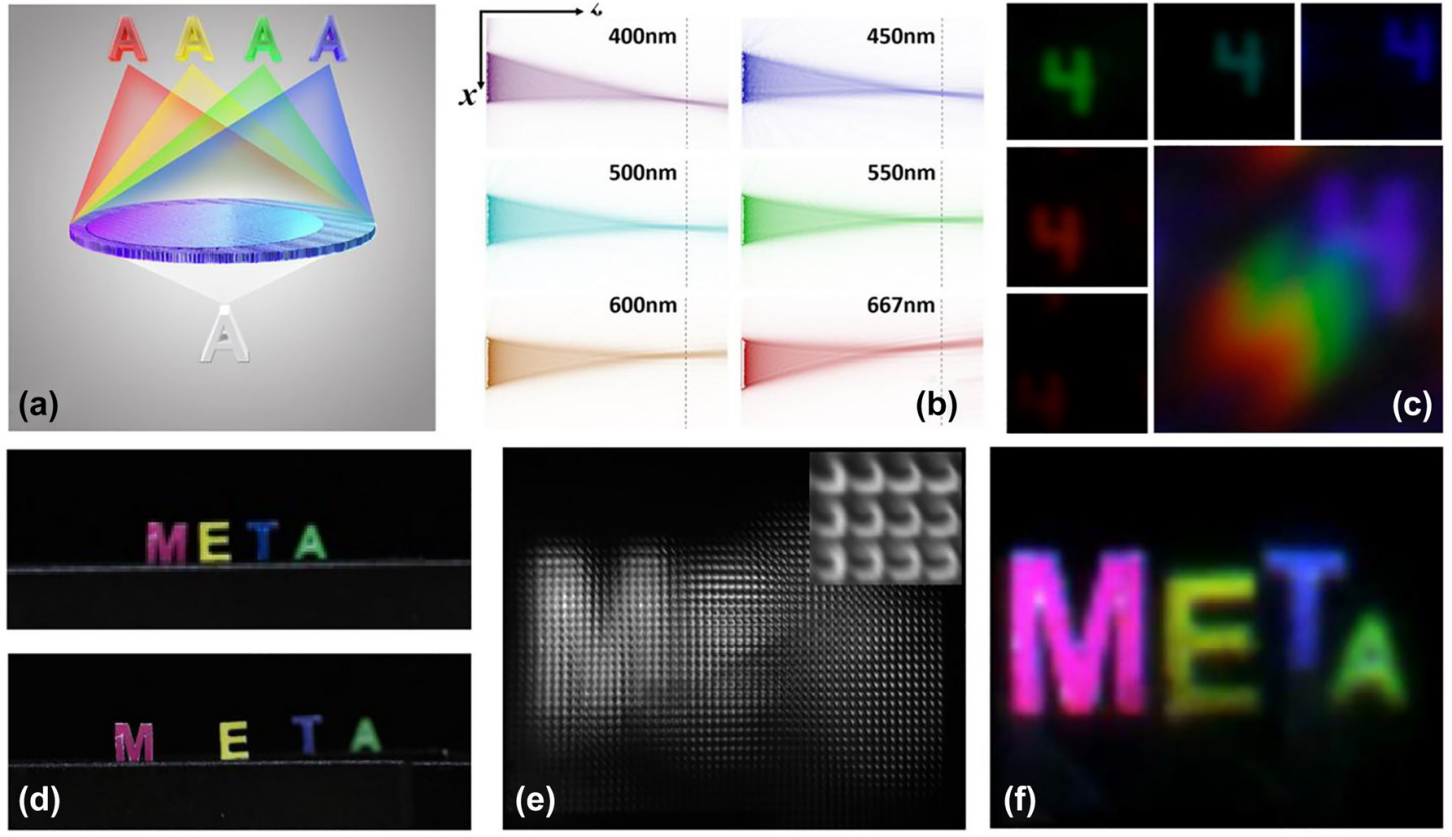
Integral field type SSI with metasurface [138]. (a) Principle of transversely dispersive metalens. (b) Simulation of the transverse distribution of focal spots at different wavelengths. (c) Image of the letter 4 by a single metalens with white light illumination. (d–f) Images of the object scene from different viewpoints. (e) Raw data acquired by detector. (f) Color image after all-focus rendering. (a–f) Reproduced with permission [138]. Copyright 2022, Nature Publishing Group.

The work in [135], [136], [137] demonstrates metasurface structures for customized dispersion, where well-designed off-axis focusing angles can be obtained for the dispersion of interest. This capability paved the way for the design of off-axis focusing metalenses with simultaneous focusing and dispersion capabilities. On this basis, Hua et al. [138] designed micro-metalenses with transverse dispersion capability [Figure 17(a–c)] and arranged them into an array to create a compact spectral light-field imaging system [Figure 17(d–f)]. This system seamlessly replaces the lenslet arrays and dispersive devices essential in traditional IFS. A single snapshot from this system can reconstruct spectral and 3D spatial information, offering a spectral resolution of 4 nm and a spatial resolution close to the diffraction limit.

Similarly, Chabot et al. [139] proposed a novel slicer concept employing a metasurface to regulate the phase on the glass substrate of the slicer stack, achieving the desired tilt of each slice. The performance of the metasurface slicing concept system was simulated and analyzed by software such as Zemax and PlanOpsim.

#### 4.1.2 Spatial replication type

**Figure 18:**
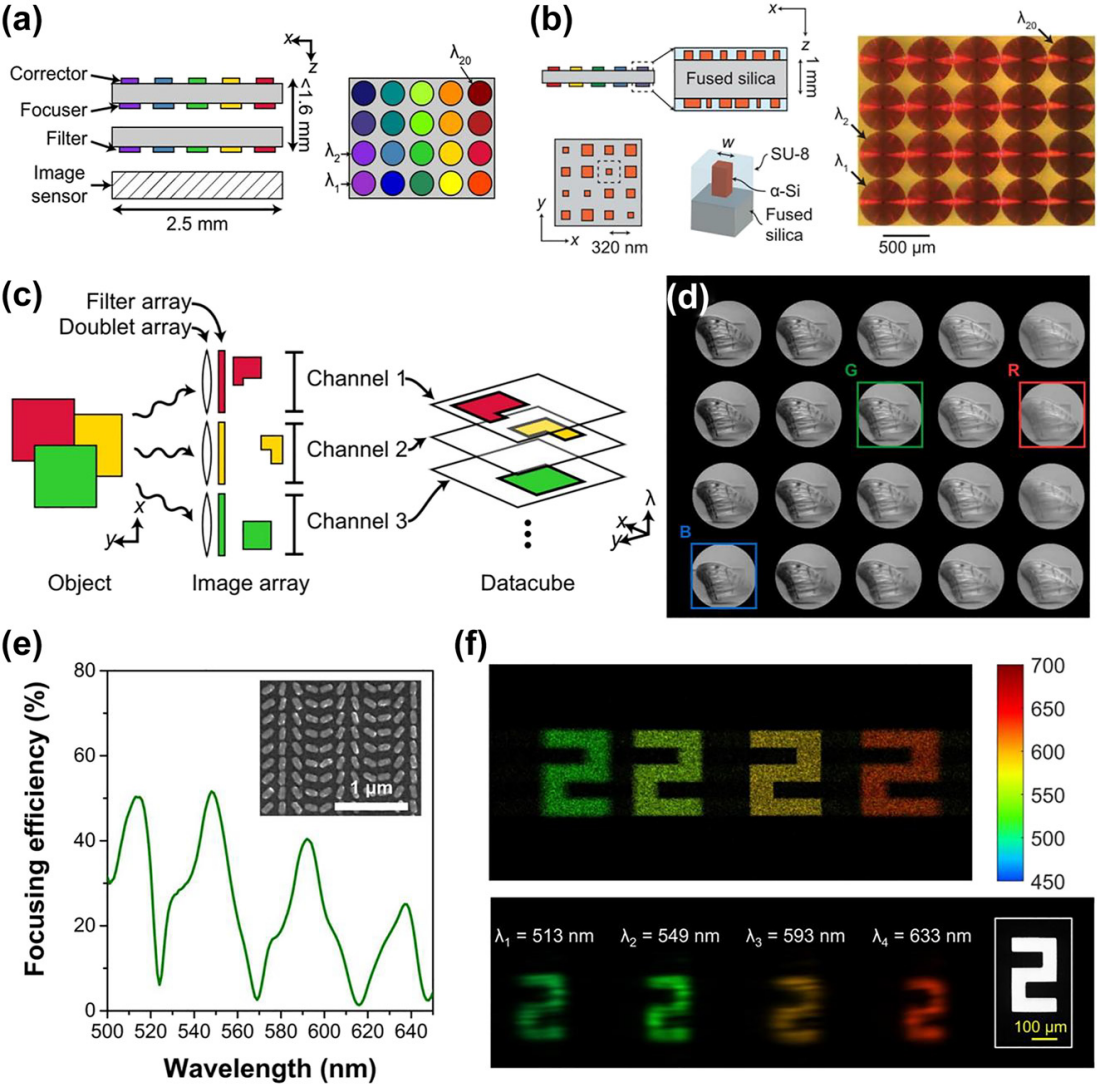
Spatial replication type SSI with metasurface [140], [141]. (a) MSSI schematic and operating principle. (b) Schematic diagram of the micro-nano structure of the metasurface. (c) Multi-channel imaging schematic. (d) Normalized image array of different spectral images. (e) The focusing efficiency of the metasurface at different wavelengths. (f) Diagram of the image captured by the detector. (a–d) Reproduced with permission [140]. Copyright 2020, American Association for the Advancement of Science. (e–f) Reproduced with permission [141]. Copyright 2023, Nature Publishing Group.

McClung et al. [140] proposed a multi-aperture parallel metasurface snapshot spectral system (MSSI), capable of 20 spectral channels in the range of 795–980 nm, as shown in Figure 18(a). Each channel system comprises of a metalens doublet and a metasurface-tuned filter. The metalens doublet, consisting of a corrector and a focuser, is composed of 485 nm-tall α-Si nanocolumns with square cross-sections, as shown in Figure 18(b and c). These elements facilitate spatial image replication and chromatic aberration correction within specific wavelength bands. The metasurface-tuned filter is a narrow-band filter with an array of super-atoms sandwiched between distributed Bragg reflectors. The filter’s center wavelength is tuned by varying the nanocolumn diameter.

Lin et al. [141] proposed a multi-wavelength off-axis focusing mirror (MOFM) to realize four-channel spectral imaging. This innovative metasurface device, constructed from multi-resonance plasma super-atoms, was subsequently combined with a small-data learning theory to realize 18-channel spectral imaging in the visible band, as shown in Figure 18(e and f).

### 4.2 Coded reconfiguration with metasurface

Empowered by efficient computational acceleration, metasurfaces can achieve nearly all forms of traditional coded aperture spectral imaging at a much lower cost, especially enabling on-chip spectral imaging to become possible. The optical field modulation realized by metasurfaces can be multifaceted. It can flexibly replace conventional coding devices to realize joint modulation. We present here the improved design and development potential of conventional systems in terms of amplitude-coded, phase-coded, and wavelength-coded, as well as the potential for development.

#### 4.2.1 Improved amplitude-coded spectrometer

Traditional CASSI systems typically achieve coded modulation by placing binary pixelated coding mask at different positions in the optical path. When combined with dispersive elements, sparse spectral imaging can be realized. However, CASSI systems are generally bulky, and the utilization of fixed coding patterns impacts the incoherence of the system matrix, thereby limiting there practical applications.

**Figure 19:**
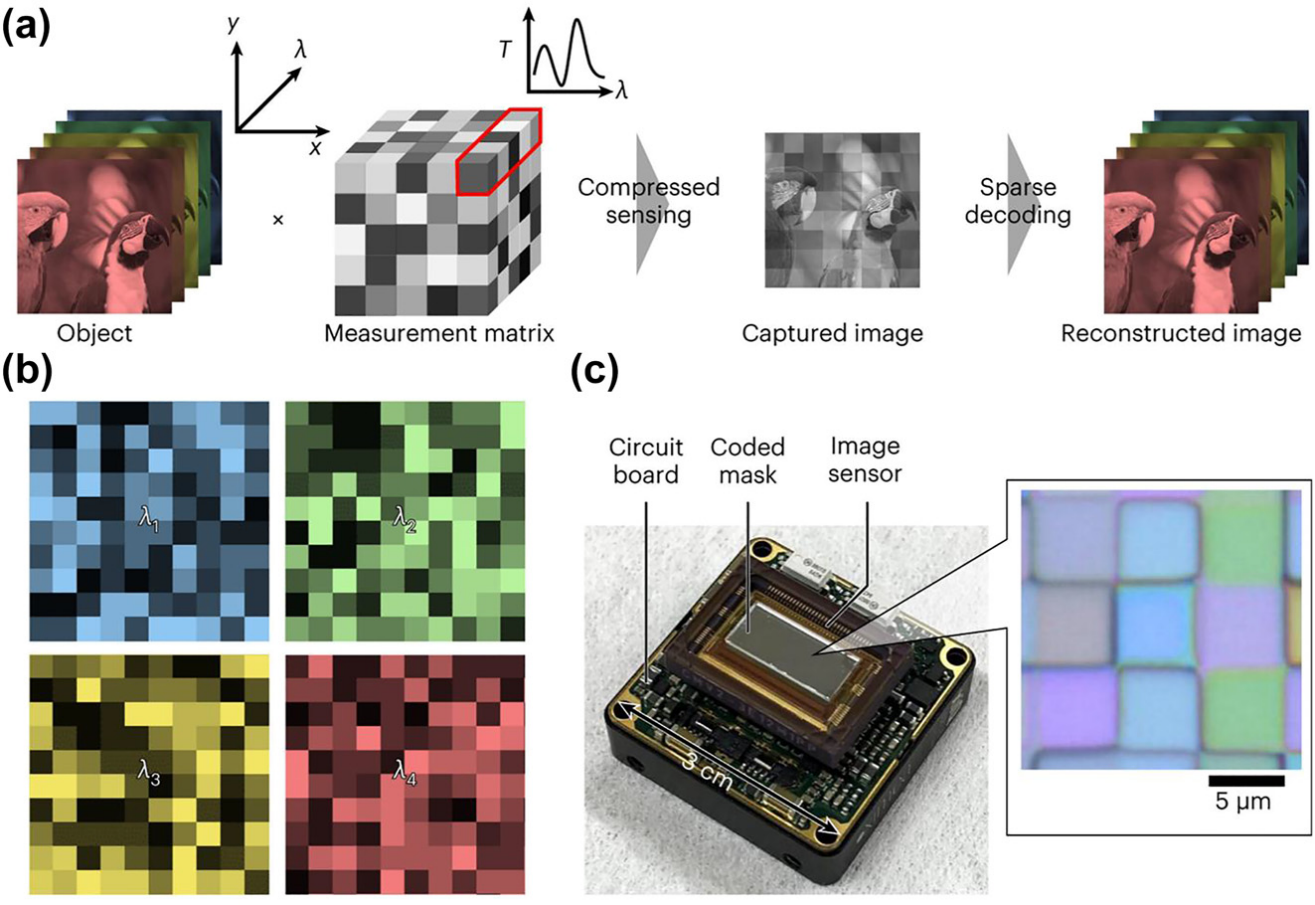
Schematic diagram of a video-rate hyperspectral camera [142]. (a) Schematic diagram of spatial-spectral coding modulation. (b) Transmission patterns of coded mask at different wavelengths. (c) Integration of detector and coded mask. (a–c) Reproduced with permission [142]. Copyright 2023, Nature Publishing Group.

The work of Yako et al. in 2023 demonstrated the realization of CASSI using a single device integrated with a detector [142], as shown in Figure 19. They used an optimally-designed F–P coding mask to achieve three-dimensional encoding. Specifically, varying the thickness of the F–P cavity produces different transmission spectra. The spectral transmittance of the mask cells enables the corresponding spectra to be gray-scale encoded, and the highly compressed images are subsequently decoded and reconstructed by the TwIST algorithm. Notably, the manufacturing difficulty of this F–P design is directly proportional to the number of transmission spectra.

Similarly, Yang et al. [143] developed a free-form metasurface-based spectral imaging chip to achieve fast spectral imaging. In contrast to those metasurface with excellent spectral filtering properties, the proposed ones are easier to fabricate.

#### 4.2.2 Improved wavelength-coded type

The great potential of novel optical metasurfaces for spectral filtering was mentioned in the previous section, and here we categorize them into two types, narrowband and broadband, for detailed elaboration.

##### 4.2.2.1 Narrowband filters array

Utilizing the interaction between micro-nanostructures and electromagnetic waves, a variety of metasurface-based narrowband spectral filters have been designed to be used as MSFAs. These micro-nanostructures can take forms such as all-dielectric resonators [144], [145], [146], hybrid plasmonic-dielectric nanostructures [147], and so on.

**Figure 20:**
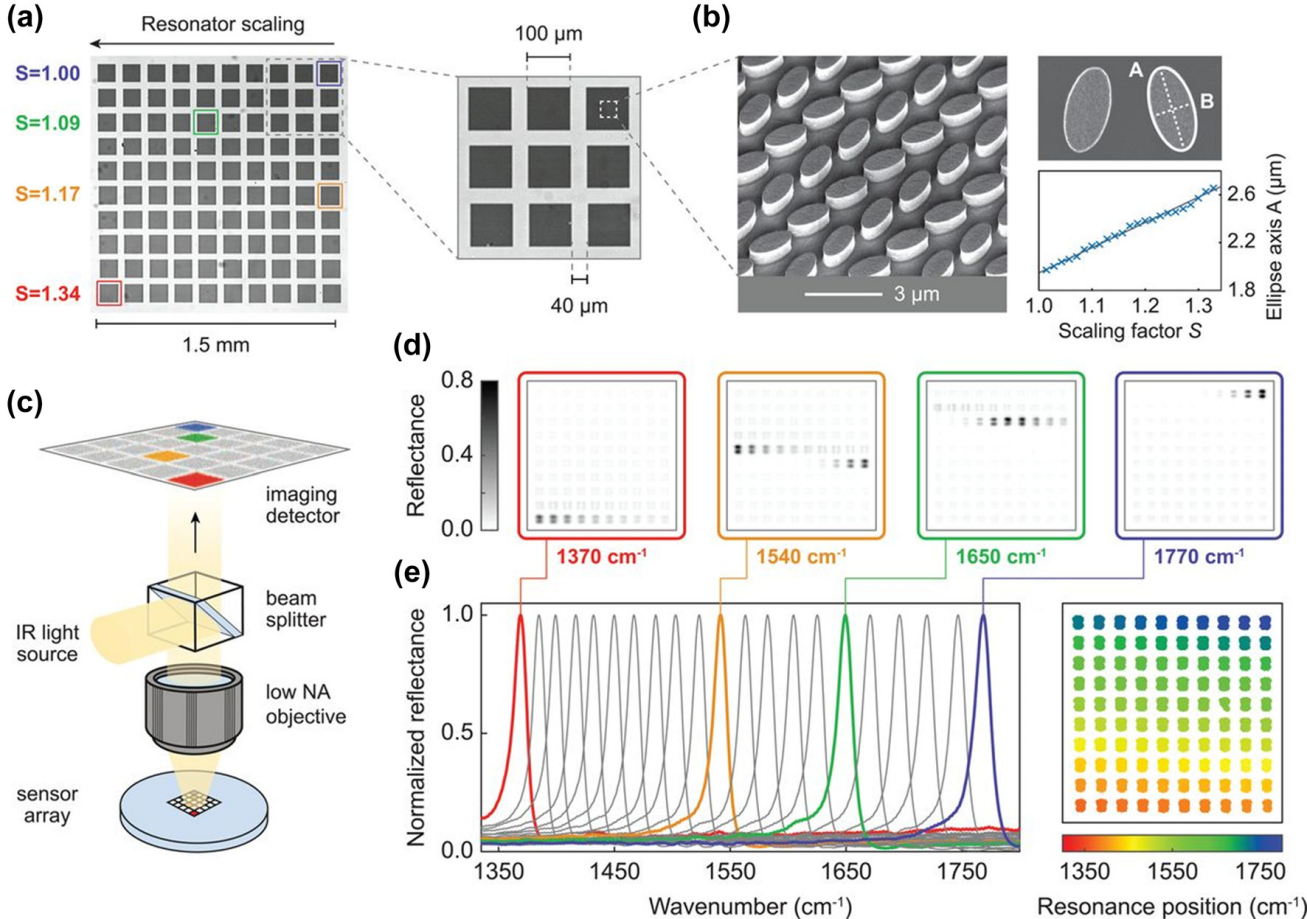
Schematic of metasurface narrowband filtering [144]. (a) Pixelated metasurface image. (b) SEM micrograph. (c) Diagram of the imaging system. (d–e) Reflection images of the pixelated metasurface recorded at four specific wave numbers. Reproduced with permission [144]. (a–e) Copyright 2018, American Association for the Advancement of Science.

These metasurface-based MSFAs can be paired with detectors to directly read out values across different spectral bands, see Figure 20. In 2018, Shaltout et al. [148] designed a compact F–P nanocavity embedded in a metasurface. Different resonant wavelengths were obtained by optimally designing the width of the metasurface structural unit, while multiple cavities allowed by the same planar chip ensured multispectral filtering capability, with the potential to be extended to on-chip spectral imaging. Lee et al. [149] integrated dielectric multilayer film filters into CMOS image sensors and adjusted the transmission wavelengths of the corresponding spectral channels by changing the size and position of the Si nanopillar arrays embedded into the respective pixel multilayer resonant cavities. Remarkably, this design obviates the need for the alignment of optical elements, and the system includes 20 channels, covering a range from 700 to 950 nm, with each spectral channel having a Full Width at Half Maximum (FWHM) of 2.0 nm.

##### 4.2.2.2 Broadband filters array

**Figure 21:**
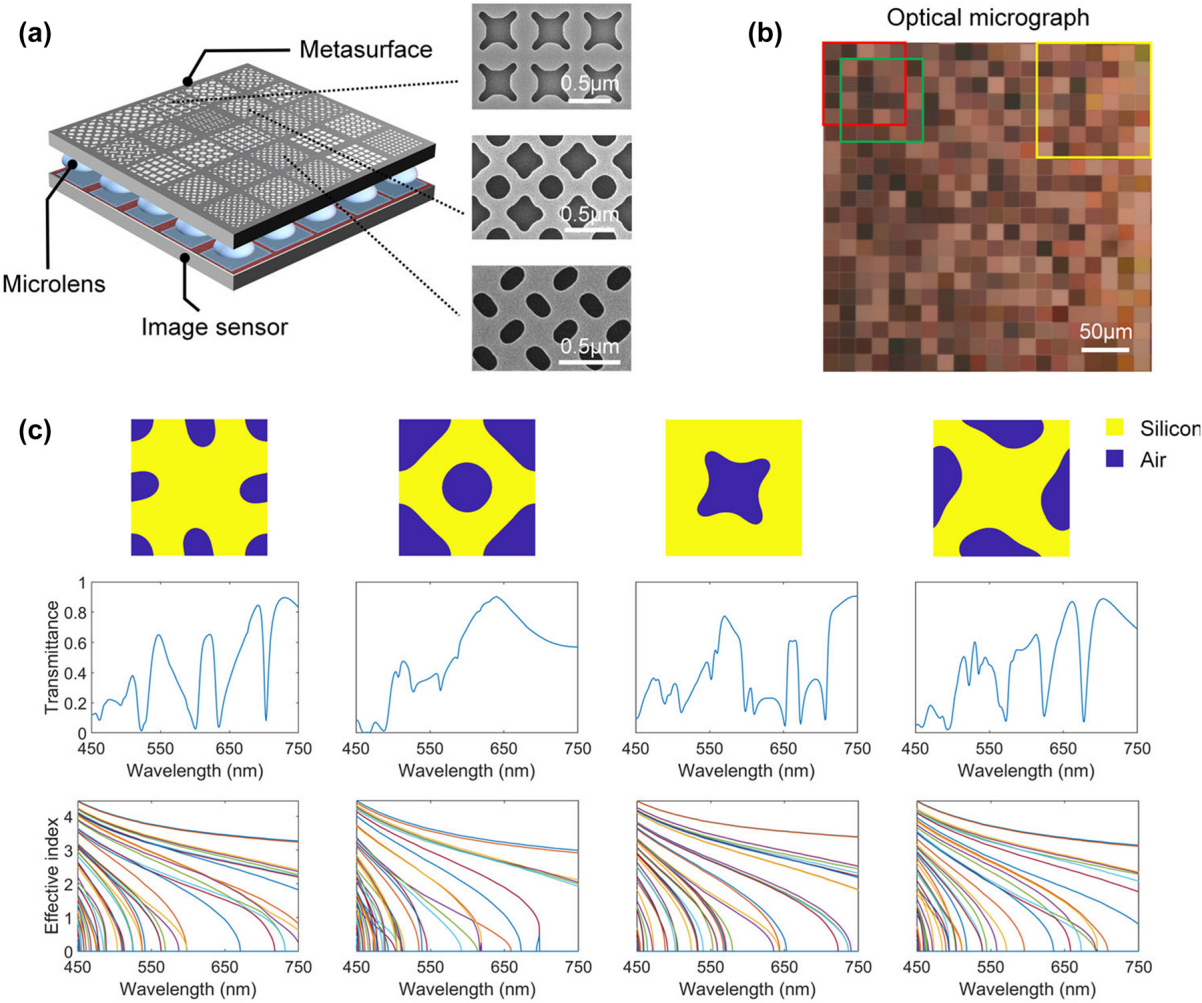
Schematic of metasurface broadband filtering [153]. (a) Diagram of the imaging system consisting of the metasurface layer, microlens layer and image sensor layer and SEM images of the three shape patterns. (b) Optical micrographs of 20 × 20 different metasurface units fabricated. A micro-spectrometer can contain 5 × 5 or 7 × 7 cells as shown in the red/green/yellow boxes. (c) Transmission spectra of four metasurface cells with free-form meta-atoms and effective indices of the Bloch mode. (a–c) Reproduced with permission [153]. Copyright 2022, Wiley-VCH.

For spectral detection, methods based on broadband random encoding have been proposed, such as quantum dot spectrometers [150], spectrometers based on photonic crystal slabs [151], and spectrometers based on metasurfaces and multilayer films [152], etc. These methods rely on the principle of wavelength multiplexing, where spectral are modulated using elements with different spectral responses, and finally the original spectral information is reconstructed from the modulated detection data. The micro-nano structures on the metasurface enable it to have a certain wavelength response over a wide spectral range, making it suitable for the fabrication of broadband MSFAs, see Figure 21.

Assuming that *K* microstructures with different spectral modulation functions $T_{i}\left(\lambda \right)$ are designed, the target spectrum $f\left(\lambda \right)$ is modulated by these elements and then detected by a monochromatic detector separately to obtain *K* measurements:

(28) $$I_{i}=\int f\left(\lambda \right)T_{i}\left(\lambda \right)\text{d}\lambda +N_{i},i=1,2,\ldots ,K,$$

where *N*_*i*_ is the noise. The measurement model can also be expressed as

(29) y=Tf+n,

where $T\in R^{L^{\prime }\times L}$ denotes the full spectral modulation function, *f* is the spectrum to be solved, and *y* is the measured value. Typically, *L*′ ≪ *L*, then Eq. (29) becomes a compressed measurement model and the original spectral can be reconstructed using a compressive reconstruction algorithm.

Wang et al. [151] presented an on-chip spectral sensor based on photonic crystal arrays in 2019, and demonstrated its capability for snapshot spectral imaging. By changing the period, size, and other parameters of the microstructures on the photonic crystals, 36 different transmission spectra were obtained, and the reconstructed spectra had a resolution of 1 nm in the range of 550–750 nm.

In 2020, Wu et al. [154] proposed a broadband random MSFA based on surface plasmonic exciton (SPP) with nine modes, where each filter consists of square holes in a square array of dots composed of a 100 nm-thick aluminum film. In 2022, Wu et al. [155] proposed a random broadband filter array based on all-dielectric gratings. In the random broadband filter array proposed by Hu et al. [152], the metasurface repeating unit of each pixel has a different shape, which in turn gives a different spectral response.

Cui and Huang et al. designed a metasurface broadband filter array with even more diverse structures [153], [156], which was subsequently developed as a real-time hyperspectral imaging chip using a deep learning-based algorithm for reconstruction, with a spectral resolution of 0.8 nm in the 450–750 nm range (0.5 nm in Ref [153]) and a spatial resolution of 356 × 436 [143]. The team’s accomplishments have reported high-precision spectral reconstruction in application scenarios such as mouse brain blood flow detection [156], face recognition [157], and autonomous driving [143].

#### 4.2.3 Improved phase-coded spectrometer

Metasurface devices can be used to replace traditional DOE elements such as conventional 2D gratings and diffractive lenses.

In 2023, Zhang et al. [158] utilized the powerful phase modulation capability of metasurfaces to design a metalens with wavelength-dependent PSFs whose phase profile consists of a fixed hyperbolic phase and an optimizable polynomial phase. Snapshot spectral imaging was achieved through the joint optimization of device design and reconstruction algorithm, demonstrating the great advantages of the metasurface over conventional diffraction spectral imaging. Zhou et al. [159] proposed an all-dielectric metamaterial array for replacing the computer-generated holographic (CGH) in order to make the CTIS more compact, which consists of two-dimensional TiO_2_ nanopillar arrays aligned on a SiO_2_ substrate. The results show that the optimized design can achieve diffraction projection of five diffraction orders while ensuring the uniformity of the spot.

## 5 Conclusions and perspectives

Snapshot Spectral Imaging (SSI) has made great progress since its introduction. This paper reviews the research progress of SSI techniques, including spatial-spectral mapping imaging techniques of integral field type and spatial replication type; amplitude modulation, wavelength modulation, phase modulation, and joint modulation techniques in coded reconstruction snapshot spectrometers; and the latest techniques involving device substitution or system integration using metasurfaces.

Compared with scanning or tunable spectral imaging methods that suffer from the “time-multiplexing dilemma,” SSI techniques offer unparalleled advantages in capturing dynamic scenes and hold promise for high spatial and spectral resolution imaging as optical fabrication and intelligent algorithms evolve.

Table 1 compares the performance parameters of each representative technology, including core devices, band range, spectral-spatial resolution, size, and spectral reconstruction speed. Obviously, while the existing array of technological solutions can cover almost all spectral ranges, achieve high-resolution imaging, and some systems can even achieve on-chip integration, the realization of systems that combines all these advantages remains challenging and has yet to emerge. It still faces the trade-offs between manufacturing difficulty, system complexity, imaging performance, and computational burden.

**Table 1:** Comparison of different snapshot spectral imaging technologies.

Type	Device	Spectral range/nm	Spectral resolution/nm	Spatial resolution/px	Size	Reconstruction speed/fps
Spatial-spectral mapping spectral imaging	Slicer mirrors	[161]: 520–660	5.6	100 × 100	Table-top	N/A
		[162]: 450–650	3.3	285 × 285	Table-top	N/A
		[13]: 480–1000	0.26	300 × 300	Room-sized	N/A
	Lenslet array	[163]: 400–760	0.74	268 × 76	Table-top	N/A
		[24]: 500–650	0.82–4.17	35 × 35–40 × 40	Table-top	N/A
	Optical fiber bundle	[30]: 450–750	4.9	188 × 170	Table-top	N/A
		[164]: 515–570	2.75	350 × 350	Table-top	N/A
		[165]: 400–1050	3.2	∼63 × 63	Table-top	N/A
		[165]: 950–5000	8	22 × 22	Table-top	N/A
	Kösters prism	[39]: 800–1700	225	–	Table-top	N/A
	Linear variable filter	[45]: 380–850	5.8	400 × 400	Hand-sized	N/A
Coded reconfiguration spectral imaging	Coding mask	[166]: 455–650	5.9	256 × 248	Table-top	30
		[167]: 3770–4800	10	640 × 480	Table-top	50
		[168]: 7,700–14,000	94	640 × 512	Table-top	50
	Filter array	[169]: 406–688	25	∼1008 × 759	On-chip	–
		[142]: 450–650	10	640 × 480; 1920 × 1080	On-chip	32.3; 7.14
[170]: 1500–1800	16	80 × 60	On-chip	–
	Diffractive optical element	[88]: 470–740	5	203 × 203	Table-top	–
[100]: 420–650	9.2	1440 × 960	Hand-sized	7.8
Metasurface-based spectral imaging	Dispersive metalens	[138]: 400–667	4	3600 × 3600	On-chip	–
	Off-axis focusing metamirror	[141]: 480–650	9.4	–	On-chip	–
	Metasurface filters array	[156]: 450–750	0.8	356 × 436	On-chip	>30
	Parallel metasystems	[140]: 795–980	9.25	∼240 × 240	On-chip	–

For spatial-spectral mapping spectrometers, which rely heavily on innovative optical designs for one-to-one mapping between spectral cube voxels and detector pixels, there is an inherent trade-off between spectral and spatial resolution, further limited by the number of detector pixels. The tunable system in [24] is a typical case where an increase in spectral resolution implies a compromise in spatial resolution. As mentioned earlier, the resolution and bandwidth of an integral field spectrometers are directly proportional to the effective dispersion optical path of the system, e.g., telescope systems in astronomical observations typically have meter-scale volumes and require cascading multiple detectors to obtain sub-nanometer spectral resolution. Spatial replication imaging spectrometers typically based on beam-splitting prisms or lenslet, although relatively compact, have a spectral resolution determined by the number of cascades of beam-splitting devices or the number of arrays of lenslet, and have difficulty in achieving high-resolution imaging. Despite these limitations, they have the significant advantage of acquiring spectral cube data of the target scene without computation, and “what you see is what you get” ensures the reliability and stability of their imaging, allowing them to be used in astronomical and microscopic imaging.

With the development of computational imaging, coded reconstruction spectrometers are expected to address the limitations of spatial-spectral mapping type SSI techniques, which are constrained by the number of detector pixels. These systems employ innovative optical devices, such as coded masks, diffractive devices, and pixel-level filter pieces, to achieve optical field modulation. These devices often require photolithography or coating processes to attain patterned micro-nano-structures or multilayer film system structures. With the emergence of technologies like laser beam direct writing and nanoimprinting, high-volume, low-cost device fabrication becomes feasible. Currently, the most mature wavelength-encoded spectrometers have been commercialized in the visible near-infrared band, enabling multi-channel, high-resolution spectral snapshot spectral imaging. However, a significant challenge faced by computational spectrometers is the requirement for an accurate system measurement matrix, especially for amplitude-encoded and phase-encoded types. This means that multiple multi-band spectral calibrations must be performed to model the imaging forward, which directly affects the upper limit of the imaging resolution of the system. In addition, the computational load is another challenge they face. The multiplexing of spatial-spectral information improves the sampling efficiency on the one hand, but it also leads to further ill-conditioning of the reconstruction model, which directly affects the quality of the reconstructed data cube; and it is difficult to apply the reconstruction model to refined observational scenarios, considering that the reconstruction model has a non-unique solution, i.e., theoretically it is difficult to make the spectral reconstruction exactly the same every time. Certainly, the development of deep learning technology enables training based on extensive data, incorporating prior knowledge into the spectral reconstruction process. This represents a crucial pathway for further enhancing the high-precision and high-resolution reconstruction of spectra.

One of the most exciting developments in SSI is the introduction and integration of metasurfaces. These techniques provide a wide range of possibilities for manipulating optical properties at sub-wavelength scales, thereby expanding the degrees of freedom to achieve optimal data acquisition conditions. Differing from traditional dispersion devices that trade increased propagation distance for higher resolution and broader bandwidth, metasurface devices have the capability to achieve efficient wide-angle super-dispersion [135], [136], [137]. This enhances the upper limit of spectral resolution. Furthermore, it significantly reduces the required dispersion optical path, thereby minimizing system dimensions. The previously mentioned off-axis dispersion-concentrating meta-lens serves as a compelling demonstration [138], [141]. Including commercially available spectral-filtering metasurfaces [160], multi-functional metasurfaces present an opportunity for creating more compact, efficient, and versatile SSI systems. Currently, the primary challenge faced by metasurfaces lies in manufacturing difficulties. For instance, the dispersive performance of a phase-dispersion meta-lens is directly proportional to its structural height, where a greater height implies a more complex manufacturing process. However, with the advancements in micro-nano manufacturing processes, the realization of large-area, high aspect ratio, and mass production techniques becomes plausible. In recent years, the explosion in computational capabilities further empowers metasurface snapshot spectral imaging. This not only significantly enhances the high-resolution imaging capabilities of metasurfaces but also facilitates the reverse design of superior metasurface structures.

In addition, some new imaging devices and techniques are also expected to address the challenges faced by SSI. Examples include perovskites or quantum dot detectors [171], [172], [173], which have the potential to resolve the resolution trade-off problem inherent in existing spectral imaging techniques due to the detector’s structural or characteristic attributes. Another avenue of exploration involves studies employing dual-frequency combs as active light sources [174], integrating ultrafast lasers [175] and multiplexing techniques to achieve temporal and spectral high-resolution SSI [176]. As for metasurface devices, some flexible or tunable metasurfaces [177], [178], [179] also opens avenues for multifunctional or high-resolution SSI. These innovations represent promising directions for advancing snapshot spectral imaging.

In summary, snapshot spectral imaging remains an evolving technology, although getting the best of both spectral resolution and spatial imaging performance is challenging. In the future, snapshot spectroscopic imaging will continue to push the limits of higher imaging dimensions [180], [181], [182], faster imaging speeds [32], [183], and higher imaging resolutions [184]. Of course, this will require novel optics, innovative optical engineering, and efficient reconstruction algorithms to achieve.
